# Forecasting carbon futures price: a hybrid method incorporating fuzzy entropy and extreme learning machine

**DOI:** 10.1007/s10479-021-04406-4

**Published:** 2021-12-30

**Authors:** Peng Chen, Andrew Vivian, Cheng Ye

**Affiliations:** 1 Department of Finance and Institute of Finance, School of Economics, Jinan University, Guangzhou, 510632 China; 2 School of Business and Economics, Loughborough University, Leicestershire, LE11 3TU UK; 3 Department of Finance, School of Economics, Jinan University, Guangzhou, 510632 China

**Keywords:** Carbon futures price, EEMD, Fuzzy entropy, K-means clustering method, ARMA, Extreme learning machine

## Abstract

In this paper, we propose a novel hybrid model that extends prior work involving ensemble empirical mode decomposition (EEMD) by using fuzzy entropy and extreme learning machine (ELM) methods. We demonstrate this 3-stage model by applying it to forecast carbon futures prices which are characterized by chaos and complexity. First, we employ the EEMD method to decompose carbon futures prices into a couple of intrinsic mode functions (IMFs) and one residue. Second, the fuzzy entropy and K-means clustering methods are used to reconstruct the IMFs and the residue to obtain three reconstructed components, specifically a high frequency series, a low frequency series, and a trend series. Third, the ARMA model is implemented for the stationary high and low frequency series, while the extreme learning machine (ELM) model is utilized for the non-stationary trend series. Finally, all the component forecasts are aggregated to form final forecasts of the carbon price for each model. The empirical results show that the proposed reconstruction algorithm can bring more than 40% improvement in prediction accuracy compared to the traditional fine-to-coarse reconstruction algorithm under the same forecasting framework. The hybrid forecasting model proposed in this paper also well captures the direction of the price changes, with strong and robust forecasting ability, which is significantly better than the single forecasting models and the other hybrid forecasting models.

## Introduction

The increasing global attention on environmental issues such as climate change in recent decades, has raised interest in understanding the topics related to it. Carbon markets are one important mechanism to tackle climate change. A major step was the establishment of the European Union Emissions Trading System (EU ETS) which has become the biggest and most important carbon exchange market in the world; as such it attracts the attention of global organizations to govern carbon emissions as well as international investors as an opportunity for the investment in this market. However, as an emerging policy-based market, the carbon market is determined by internal market mechanism (such as the quantity of allowances issued) as well as external factors (such as macroeconomic factors; Zhang and Wei, 2010). The determinants together lead to strong fluctuations in the carbon market, which is characterized by chaos and high volatility with nonlinear, non-stationary phenomena evident in its prices. Further understanding the patterns of price fluctuations and predicting movements more accurately is of great importance to practitioners, policymakers and academics. For policymakers’ it will be useful to gauge when the supply of carbon permits needs adjusting to avoid prices moving to extreme levels.^1^ For investors it is critical to know how the price is likely to move so that they can exploit this in their trading decisions. Moreover, from an academic perspective, our paper contributes to the literature by demonstrating that a new hybrid method that features fuzzy entropy and extreme learning machine is effective in forecasting carbon futures price.^2^

Over the last decade, a growing body of empirical studies on the forecasting of carbon prices has emerged (Byun and Cho, 2013; Fan et al., 2015; Zhu et al., 2018; Song et al., 2019). In earlier studies, traditional statistical and econometric models, such as linear regression, ARMA models and VAR models have been widely applied to carbon prices forecasting. The models achieve a desirable prediction performance when the prices series is linear or near-linear. However, the dynamics of carbon futures prices series is characterized by the features of nonlinearity and irregularity (Arouri et al., 2012). Due to the limitations of traditional econometric models on the nonlinear pattern of price series, recent scholars turn to some emerging nonlinear artificial intelligence (AI) models or statistical learning, such as artificial neural network (ANN), support vector machines (SVM) and least squares support vector machines (LSSVM), to forecast carbon prices. Many experiments have demonstrated the superiority in carbon price prediction of AI-based models when compared to traditional statistical models (e.g., Tsai and Kuo, 2013; Fan et al., 2015; Jiang and Wu, 2015). Although the AI-based models have strong prediction ability, they have their own drawbacks in that they are very sensitive to parameters selection, which may often lead to long training time or no convergence. To remedy these shortcomings, the extreme learning machine (ELM) proposed by Huang et al. (2006) has recently been applied to time series prediction (Sun and Zhang, 2017).

Moreover, some hybrid models have been proposed to forecast carbon futures prices and achieve a better performance. For example, Zhu and Wei (2013) develop a novel hybrid method integrating the ARIMA with LSSVM model to forecasting carbon prices. Zhang et al. (2017) propose a hybrid model that combines signal processing technology, econometric models and neural network for carbon prices forecasting. Huang et al. (2021) combine econometric and neural network methods to propose a novel decomposition-ensemble paradigm VMD-GARCH/LSTM-LSTM model for carbon price forecasting, providing evidence of superiority in forecasting accuracy.

In addition, empirical mode decomposition (EMD) method proposed by Huang et al. (1997), which can not only automatically determine the total decomposed number of the original signal, but also can extract linear stationary components from nonlinear and non-stationary original time series, has also been widely used to forecast time series (Guo et al., 2012; Jiang et al., 2020; Wang, et al., 2016; Zhang et al., 2008), including carbon price (Zhu, 2012; Zhu et al., 2017, 2018, 2019); these features help explain why it has been (recently) preferred to wavelet decomposition. Prior work has used EMD along with other methods such as the AI-based models and contributed by demonstrating it is effective for forecasting (Zhu et al., 2017).

Motivated by hybrid methods and the “decomposition-and-ensemble” principle, the paper proposes a new hybrid method for forecasting carbon futures prices. This method, incorporates the ensemble EMD, and introduces i) Fuzzy entropy and ii) extreme learning machine (ELM) into the model. This leads to a novel hybrid multiscale nonlinear ensemble ELM approach to forecasting carbon futures prices. The advantages of the novel hybrid approach to forecasting are as follows. First, compared with other popular forecasting methods, this novel hybrid framework provides more accurate and efficient prediction of the nonstationary and nonlinear carbon futures prices. Second, this paper is the first to use the fuzzy entropy analysis and K-means clustering method to reconstruct the IMFs and the residue decomposed by the EEMD; the approach adopted in this paper is therefore truly novel. Compared to traditional fine-to-coarse reconstruction algorithm that ignores the complexity of the decomposed IMFs as well as directly using the residue as the trend component, the proposed reconstruction algorithm (using the Fuzzy Entropy and K-means clustering methods) under the decomposition-ensemble framework is more efficient and accurate to reconstruct the three components, (i.e., i) high frequency, ii) low frequency and iii) trend). Third, the proposed hybrid model makes full use of the advantages of econometric model as well as artificial intelligence model in that the ARMA and the ELM forecasting model are established for the stationary and non-stationary components, respectively. Compared to the traditional methods (such as Back-Propagation method) in dealing with the non-stationary trend series, the Extreme Learning Machine (ELM) works efficiently without iteration. In addition to working efficiently, the proposed method is easily processed and requires less sample for the training set than many alternatives. Finally, this paper first proposes a time-varying cross-validation method to select the optimal parameters for the ELM model. This is due to the fact that there is short-term memory in the carbon futures prices since the series is not a random walk (Feng et al., 2011).

The remainder of the paper is organized as follows: Sect. 2 gives a brief review of the related literature. Section 3 describes the proposed novel hybrid forecasting model and outlines the main integrated modules, including EEMD, fuzzy entropy, extreme learning machine (ELM). Section 4 outlines the measures of forecast errors and describes the data; Sect. 5 provides empirical analysis; Sect. 6 presents forecast results; Sect. 7 concludes.

## Literature review

Many different aspects of the carbon market have been examined by the rapidly developing literature. Montagnoli and De Vries (2010) provide early evidence of trading thickness and efficiency of the European carbon market during phase I (2005–2007) and the beginning of phase II (2008–2009). They find that during phase I the market is characterized by inefficiency but during phase II it is becomes increasingly efficient. Feng et al. (2012) provide early evidence on tail risk in the European carbon market using extreme value theory to effectively estimate value at risk; their main results indicate that downside risk is high in the market and it is of greater magnitude than upside risk. Relatedly, Jiao et al. (2018) emphasise the important role that macroeconomic fundamentals can play in enhancing value at risk estimates. Rootzén and Johnsson (2016) take a different perspective and looks at the impact of the carbon price on car production (especially steel cost) finding that even a relatively high carbon price (100€/tCO_2_) has a very modest impact on the cost of a car. In contrast, Zhang et al. (2016) project carbon emissions in China under various scenarios finding that carbon emissions can be reduced at modest cost by 2050. Recently, Tan et al. (2020) examine how connected the European carbon market is to energy and financial markets; they find that carbon is connected more closely to energy markets than to equity and bond markets.

Earlier studies in the literature have widely employed traditional statistical and econometric models for the forecasting of carbon prices and volatility. For example, Byun and Cho (2013) employ the GARCH family of models to forecast carbon futures volatility and find that GJR-GARCH is more effective than TGARCH and GARCH in prediction. When using the AR–GARCH and regime-switching models for stochastic modeling the price dynamics of CO_2_ emission allowances, Benz and Trück (2009) find that these models adequately capture characteristics like skewness, excess kurtosis and in particular different phases of volatility behavior in the returns. Sanin et al. (2015) incorporate a time-varying jump probability to the hybrid ARMAX-GARCH model and find that this model obtains a better performance of carbon volatility prediction than the standard ARMAX-GARCH framework does. Although GARCH and Markov switching multifractal (MSM) models might capture different facets of the volatility process in carbon prices, Segnon et al. (2017) find that MSMs in most cases encompass GARCH and FIGARCH when comparing the volatility forecasting between different models. Chevallier (2011) proposes a nonparametric modeling approach to the carbon prices and volatility showing that it reduces the prediction error for conditional mean by almost 15% compared to linear AR models. The above econometric models can produce high prediction accuracy when the data used in the studies is linear or stationary. In fact, carbon price is a time series with nonlinear, non-stationary, chaotic and multifractal characteristics (Feng et al., 2011; Zhu et al., 2017), so implementing traditional econometric models may no longer be the most suitable approach for forecasting carbon prices and may lead to a larger forecasting error.

Due to the limitations of econometric models, some scholars investigate the usefulness of nonlinear artificial intelligence (AI) models or statistical learning for carbon price forecasting, such as artificial neural network (ANN), support vector machines (SVM), and least squares support vector machines (LSSVM), among other. Among the ANN models, the multi-layer perceptron (MLP) model is the most widely used in time series prediction (de Oliveira et al., 2013). For example, when using the multi-layer perceptron (MLP) model to predict the carbon prices, Fan et al. (2015) find the model possesses good prediction performance in both level and directional measurement. Although the ANN method has a strong prediction power, it highly depends on the network structure (topology, connections, neurons number) and their operational parameters (learning rate, momentum, etc.). Thus, there exist some drawbacks in the models, including long training time to get an ANN network, no convergence of the optimization algorithm, and easily falling into the local optimal solution. However, the support vector machine model (SVM) proposed by Vapnik (1995) can avoid the issue of falling into the local optimal solution and obtain the globally optimal solution, which has been widely used in time series prediction. For example, Zhu and Wei (2013) use the least square support vector machines (LSSVM) method to predict carbon prices, and show that the prediction performance is better than ARIMA and ANN do. When proposing a hybrid ARMA and SVM models to predict the carbon price, Zhu and Wei (2013) find that the hybrid forecasting model can improve the prediction accuracy.

Although the SVM model can obtain a better forecasting power, it spends a lot of time on training process and needs to determine the hyper-parameters. To cope with the above limitations, Huang et al. (2006) propose the extreme learning machine (ELM) based on a single hidden layer feedforward neural network, in which the algorithm randomly generates the connection weights between the input layer and the hidden layer, and the threshold of the neurons in the hidden layer. This method not only succeeds in overcoming the drawback of long training time of the ANN and SVM models, but also can avoid falling into the local optimal solution and thus quickly find the optimal solution. Therefore, the ELM has been applied to time series prediction in various fields recently including rainfall (Taormina and Chau, 2015) and CO2 emissions (Sun, Wang and Zhang, 2017).

In fact, carbon futures price is a chaotic and highly volatile time series featuring nonlinearity, non-stationarity as well as mixed frequency characteristics. However, traditional econometric models such as the ARMA model, GARCH, and VAR methods are primarily suitable for the prediction of stationary time series, so their predictive power is very limited for non-stationary carbon price series. Further, for the single statistical learning models, such as ANN, SVM and ELM model, although they are very suitable for modelling nonlinear time series, their prediction power is also limited because the carbon price is characterized by components with different frequencies.

To remedy the above limitations, hybrid methods have been proposed for forecasting carbon prices as well as other time series in the literature (e.g., Liu et al., 2012; Liu and Shi, 2–13; Zhu and Wei, 2013; Li et al., 2017; Zhang et al., 2017; Huang et al., 2021). For example, Zhu and Wei (2013) use the ARMA-LSSVM model to predict the carbon prices and find that these combined forecasting models can achieve a better forecasting performance than a single forecasting model. However, there are some characteristics of mixed frequency (high and low) components in carbon price series, so that the prediction power of the above-combined forecasting model may be incomplete, imperfect or limited. Therefore, some forecasting methods based on decomposition-reconstruction principle are proposed for time series prediction, such as wavelet decomposition (Meng et al., 2016; Yu et al., 2015). However, the wavelet decomposition method requires one to determine the number of decomposed layers and wavelet functions in advance. In contrast, the empirical mode decomposition (i.e., EMD) method proposed by Huang et al. (1997), can not only automatically determine the total decomposed number of the original signal, but can also extract linear stationary components from nonlinear and non-stationary original time series. Consequently, this method has been widely used to forecast time series (Guo et al., 2012; Wang, et al., 2016; Zhang et al., 2008), including carbon price prediction (Zhu, 2012; Zhu et al., 2017, 2018) and carbon volatility (Tang et al., 2017). For example, Zhu et al. (2018) use a novel multiscale nonlinear ensemble leaning paradigm incorporating EMD and LSSVM with kernel function prototype for carbon price forecasting and demonstrate that the proposed model can obtain higher level and directional predictions and higher robustness. However, there exists the mode mixing problem in the EMD method. Further, an ensemble empirical mode decomposition (EEMD), proposed by Wu and Huang (2009) for solving the mode mixing problem, has been used in time series prediction (Ren et al., 2015; Wang et al., 2015; Zhang et al., 2017). For example, Zhu et al. (2017) use an EEMD-based LSSVM to predict the carbon prices, and demonstrate that the proposed model achieves high accuracy both in level and directional predictions.

## Methodology

### Ensemble empirical mode decomposition (EMD)

The method of empirical mode decomposition (i.e., EMD) proposed by Huang et al. (1997) has be widely used in the literature to decompose a nonlinear and non-stationary time series into a set of intrinsic mode functions (IMFs) and a residue.^3^ However, one major limitation is the mode mixing (i.e., the occurrence of disparate scales across different IMFs) in the EMD decomposition. To address the problem of mode mixing, Wu and Huang (2009) combine EMD with noise-assisted analysis method to propose the ensemble EMD (EEMD) method. The basic principle of the EEMD algorithm is that it defines the true IMF components as the mean of an ensemble of trials; each trial consists of the signal plus a Gaussian white noise term. The EEMD method is defined as follows:

*Step 1*: Setting the total number of white noises added is *M*, and the standard deviation of white noise is generally 0.1 ~ 0.4 times of the standard deviation of the original signal. And add the Gaussian white noise $n_{i} (t)$ with zero mean and constant standard deviation in the original signal $x(t)$.

1 $$ x_{i} (t) = x(t) + n_{i} (t)$$

*Step 2*: Decompose $x(t)$ into several IMF components and a residue by the EMD method, and the *imf*_*ij*_*(t)* is the *j*th IMF component after adding the *i*th Gaussian white noise.

*Step 3*: Repeat steps (2), (3) *M* times, and then calculate the average value of all the corresponding IMF components to eliminate the effect of the white noise added and obtain the final IMF component:

2 $$ imf_{j} (t) = \frac{1}{M}\sum\limits_{i = 1}^{M} {imf_{ij} (t)}$$

where $imf_{j}$ is the *j-th* component decomposed by the EEMD method.

### Fuzzy entropy

Entropy is a property of the thermodynamic system that can measure the disorder in the dynamic states of time series. Due to the advantage that entropy can be visualized, different entropy methods have been proposed to analyze the complexity characteristics of time series (Chen et al., 2009), including for quantifying the efficiency of financial markets (Ortiz-Cruz et al., 2012). In particular, entropy can quantitatively estimate the complexity of hidden patterns in data, but in fact, the boundaries between patterns are ambiguous and difficult to determine the relationship between patterns, thus the fuzzy entropy method based on fuzzy theory was proposed to calculate the fuzzy similarity between different hidden patterns by using membership function. Therefore, the similarity of two time series can be measured by the fuzzy entropy method. For a given time series *x(t)*, *t* = *1,2,…,T,* where *T* is the length of *x(t)*, and then the fuzzy entropy can be calculated as follows:

Step 1: For a given time series *x(t)*, the pattern dimension is set to *m*, and then the *m*-dimension vector is defined as:

3 $$ X_{i}^{m} { = }[x(i),x(i + 1),...,x(i + m - 1)],1 \le i \le T - m + 1$$

Step 2: Compute the distance between vector X(i) and X(j):

4 $$ \begin{aligned} d_{{_{ij} }}^{m} &= d[X_{i}^{m} ,X_{j}^{{\text{m}}} ] = \mathop {\max }\limits_{k = 0,1,...,m - 1} \{ \left| {[x(i + k) - x0(i)] - [x(j + k) - x0(j)]} \right|\}  \\ i,j &= 1,2,...,T - m,i \ne j  \\ \end{aligned} $$

Step 3: Given parameter *n* and tolerance parameter *r*, then the similarity $D_{ij}^{m}$ between $X_{i}^{m}$ and $X_{j}^{m}$ is estimated by the fuzzy membership function $\mu (d_{ij}^{m} ,n,r)$, i.e.,

5 $$ D_{{_{ij} }}^{m} = \mu (d_{{_{ij} }}^{m} ,n,r) = \exp ( - (d_{{_{ij} }}^{m} /r)^{n} )$$

Step 4: Compute the following function after calculating the similarity $D{_{ij}^{m}}$:

6 $$ \varphi^{m} (n,r) = \frac{1}{T - m + 1}\sum\limits_{i = 1}^{T - m + 1} {\left(\frac{1}{T - m}\sum\limits_{j = 1,i \ne j}^{T - m + 1} {D_{ij}^{m} } \right)} $$

Similarly, for $$X_{i}^{{\text{m + 1}}}$$:

7 $$ \varphi^{m + 1} (n,r) = \frac{1}{T - m}\sum\limits_{i = 1}^{T - m} {\left(\frac{1}{T - m - 1}\sum\limits_{j = 1,i \ne j}^{T - m} {D_{ij}^{m + 1} } \right)} $$

Step 5: The final fuzzy entropy of time series $x(t)$ can be calculated as follows:

8 $$ FuzzyEn(m,n,r,T) = \ln \varphi^{m} (n,r) - \ln \varphi^{m + 1} (n,r)$$

In the above Eq. (8), *m* is the pattern dimension, *r* is tolerance parameter, and *T* is the length of series. Following the method in Chen et al. (2007), the embedded dimension m usually takes 2 or 3. And *r* represents the width of the boundary of the fuzzy function, and will lose a lot of statistical information when *r* is set too large. Therefore, *r* is generally 0.1 ~ 0.25 $\sigma_{SD}$, where $\sigma_{SD}$ is the standard deviation of the original series, and n take 2 or 3.

### Extreme learning machine (ELM)

The extreme learning machine (ELM), proposed by Huang et al. (2006), is based on single-hidden layer feedforward neural networks. The algorithm randomly generates the connection weight matrix between input and hidden layers and the threshold of neurons in the hidden layer, and there is no need to adjust the connection weight and the threshold during the training process of network. The optimal solution can be achieved as long as the number of neurons in the hidden layer is determined. Compared with the traditional neural network model based on gradient descent method, the advantage of the ELM method is that it does not require iteration to obtain the optimal solution, which leads to faster learning speed and better generalization performance.

**Fig. 1:**
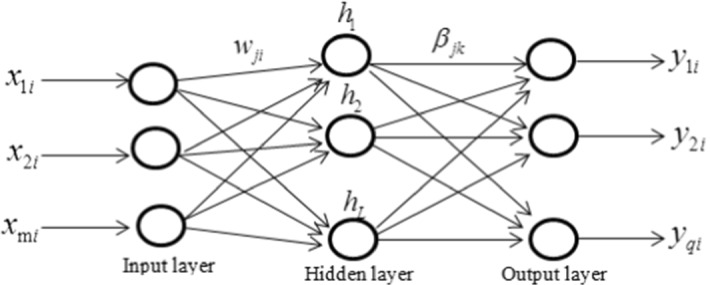
The network of extreme learning machine (ELM)

For given *n* different samples ($x_{i}$,$t_{i}$), where $x_{i} \in R^{m}$, $t_{i} \in R^{q}$ are *m, q* dimension vectors, respectively. $x_{i} = (x_{1i} ,x_{2i} , \ldots x_{mi} )^{T} ,t_{i} = (t_{1i} ,t_{2i} , \ldots ,t_{qi} )^{T}$, and the total number of neurons of the hidden layer neurons is *L*. The topology of ELM network is shown in Fig. 1.

Define the connection weight matrix between the input and the hidden layers as *W*, the threshold of neurons in the hidden layer as *b*, and the connection weight between the hidden and the output layers as $\beta$.

9 $$ w = \left[ {\begin{array}{*{20}c} {w_{11} } & {w_{12} } & \cdots & {w_{1m} } \\ {w_{21} } & {w_{22} } & \cdots & {w_{2m} } \\ \vdots & \vdots & \cdots & \vdots \\ {w_{L1} } & {w_{L2} } & \cdots & {w_{Lm} } \\ \end{array} } \right],{\text{b}} = \left[ {\begin{array}{*{20}c} {b_{1} } \\ {b_{2} } \\ \vdots \\ {b_{L} } \\ \end{array} } \right],\beta = \left[ {\begin{array}{*{20}c} {\beta_{11} } & {\beta_{12} } & \cdots & {\beta_{1q} } \\ {\beta_{21} } & {\beta_{22} } & \cdots & {\beta_{2q} } \\ \vdots & \vdots & \cdots & \vdots \\ {\beta_{L1} } & {\beta_{L2} } & \cdots & {\beta_{Lq} } \\ \end{array} } \right] $$

where $w_{ji}$ is the connection weight between the *i*th neuron of the input layer and the *j*th neuron of the hidden layer; *b*_*j*_ represents the threshold of *j*th neuron in the hidden layer; and $\beta_{jk}$ represents the connection weight between the *j*th neuron of the hidden layer and the *k*th neuron of the output layer.

Then the input matrix X and the output layer matrix Y are defined as follows:

10 $$ {\text{X}} = \left[ {\begin{array}{*{20}c} {x_{11} } & {x_{12} } & \cdots & {x_{1n} } \\ {x_{21} } & {x_{22} } & \cdots & {x_{2n} } \\ \vdots & \vdots & \cdots & \vdots \\ {x_{m1} } & {x_{m2} } & \cdots & {x_{mn} } \\ \end{array} } \right];Y = \left[ {\begin{array}{*{20}c} {y_{11} } & {y_{21} } & \cdots & {y_{q1} } \\ {y_{12} } & {y_{22} } & \cdots & {y_{q2} } \\ \vdots & \vdots & \cdots & \vdots \\ {y_{1n} } & {y_{2n} } & \cdots & {y_{qn} } \\ \end{array} } \right]$$

The activated function of the neurons in the hidden layer is set as $G(x)$, thus the output matrix of the hidden layer is calculated by

11 $$ {\text{H}} = \left[ {\begin{array}{*{20}c} {G(w_{1} x_{1} + b_{1} )} & {G(w_{2} x_{1} + b_{2} )} & \cdots & {G(w_{L} x_{1} + b_{L} )} \\ {G(w_{1} x_{2} + b_{1} )} & {G(w_{2} x_{2} + b_{2} )} & \cdots & {G(w_{L} x_{2} + b_{L} )} \\ \vdots & \vdots & \cdots & \vdots \\ {G(w_{1} x_{{\text{n}}} + b_{1} )} & {G(w_{2} x_{n} + b_{2} )} & \cdots & {G(w_{L} x_{{\text{n}}} + b_{L} )} \\ \end{array} } \right] $$

In Eq. (11), $w_{i} = (w_{i1} ,w_{i2} , \cdots ,w_{im} )$ represents the connection weight between the hidden layer and the input layer. Finally, the output matrix is calculated as:

12 $$ {\text{Y}} = H\beta$$

When the activated function $G(x)$ is infinitely differentiable, not all the parameters of the single layer feedforward neural network (*SLFN*) need to be adjusted. Here, *W* and *b* can be selected randomly before the process of training, and remains unchanged during the process of training. Therefore, the training time of the ELM algorithm is shorter than that of the BP algorithm. The connection weight $\beta$ between the hidden and the output layers can be obtained by solving the least squares of the following equation:

13 $$ \mathop {\min }\limits_{\beta } \left\| {H\beta - T^{^{\prime}} } \right\|$$

Then, the solution is $\hat{\beta } = H^{ + } T^{^{\prime}}$, where $H^{ + }$ is Moore–Penrose generalized inverse of the output matrix in the hidden layer.

### The proposed EEMD-FuzzyEn–ARMA-ELM hybrid forecasting model

Due to the non-stationary and nonlinear properties of carbon futures prices, a single ARMA model cannot fit the non-stationary carbon price series well. The hybrid model under the decomposition-ensemble framework is proposed to separate the original carbon prices into linear and stationary component, and nonlinear and nonstationary trend. In order to take advantage of the ARMA model’s prediction power on stationary time series as well as the ELM model on nonstationary time series, the proposed hybrid model incorporating the ARMA and ELM model, namely, EEMD-FuzzyEn–ARMA-ELM, are established for the linear stationary components and non-stationary components, respectively. Thus, the proposed hybrid model makes full use of the advantages of econometric model as well as artificial intelligence model for forecasting.

**Fig. 2:**
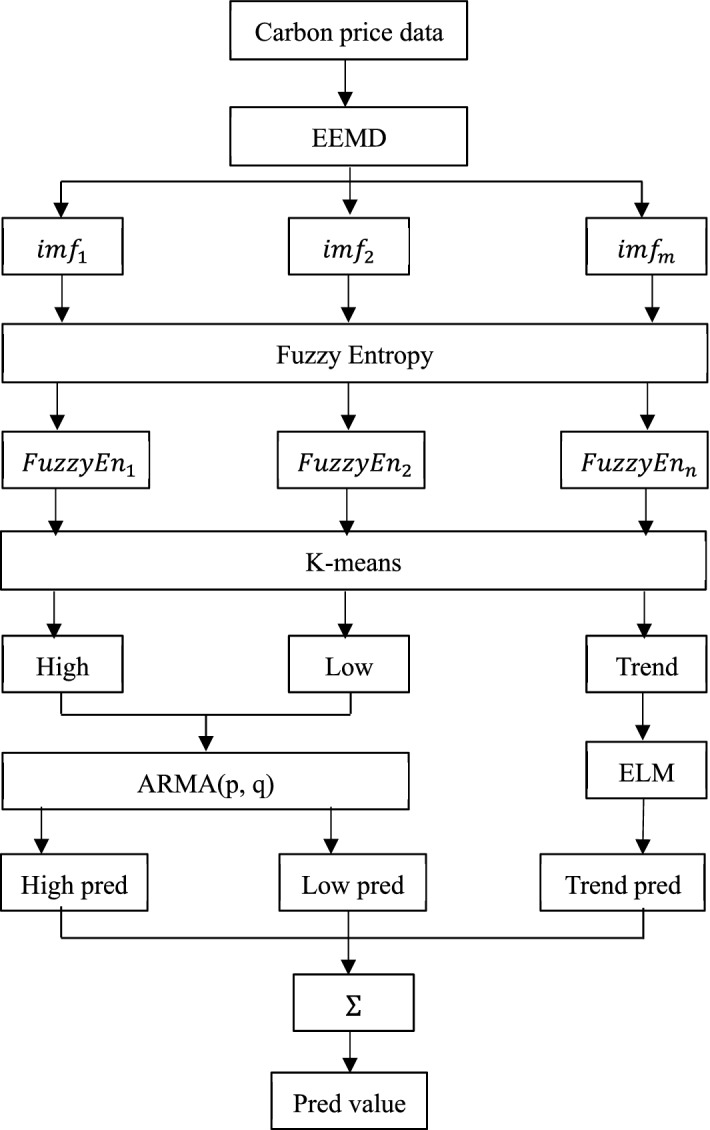
The overall framework of the proposed hybrid model

The overall framework of the hybrid forecasting model is shown in Fig. 2. The main steps are as follows:

*Step 1* : Use EEMD method to decompose carbon price into a series of intrinsic mode functions (IMFs) and a residue.

*Step 2*: Calculate the fuzzy entropy values of each IMF and the residue, which are used as the clustering feature variables. Further, the k-means clustering method is used to classify the decomposed IMFs and the residue into different clusters. Following the literature of the reconstruction of decomposed IMFs, the components are generally reconstructed into three different sequences (i.e., three clusters), including the high frequency (*High*), the low frequency (*Low*) and the trend series (*Trend*). As a result, three different series are reconstructed from the original carbon price.

*Step 3*: The stability of these reconstructed components, including the high frequency, low frequency and trend series, is examined via the unit root test. Then the ARMA and ELM models are established for the stationary high and low frequency series, and non-stationary trend series respectively.

Step 4: The predicted results of the high-frequency (*High*), low-frequency (*Low*) and trend series (*Trend*) based on ARMA model and ELM model are denoted as *High_pred*, *Low_pred*, and *Trend_pred*, respectively, and then the final prediction of the original time series based on the hybrid forecasting model is obtained by aggregating *High_pred*, *Low_pred*, and *Trend_pred*, i.e.,

14 $$ pred\_value \, = \, High\_pred + Low\_pred + Trend\_pred$$

In sum, this study employs the EEMD method to decompose the nonlinear and non-stationary carbon futures prices, which effectively separate the stationary and nonstationary components. In order to reduce the training time and the number of elements requiring training for the forecasting model, fuzzy entropy and K-means clustering methods are used to reconstruct the IMFs and the residue. We obtain three different frequency series, including the high frequency series representing the carbon market fluctuation component, the low-frequency series representing the major event-driven component, and the trend series representing the tendency of the carbon prices respectively. Further, we take advantages of the prediction power of the ARMA model as well as the ELM model, in which the ARMA model is established for the stationary high and low frequency series, while the extreme learning machine (ELM) model is established for the non-stationary trend series. Finally, the forecasting results of all the components based on the forecasting models are aggregated as the final forecasting of the carbon prices.

## Data and evaluation criteria

### Evaluation criteria

In order to evaluate the predication performance of the models, we use four different measures of forecast error, including root mean square error (RMSE), mean absolute percentage error (MAPE), correlation coefficient (R) and direction statistic (DS), as the evaluation criteria.^4^

Moreover, we implement the adjusted Diebold-Mariano test (i.e., DM, there) proposed by Harvey et al. (1997) to evaluate the predictive accuracy between different forecast models. When forecast errors follow heavy-tailed distributions, the test framework in the seminal paper by Diebold and Mariano (1995) is found to be quite seriously over-sized, which may generate some misleading finding. The adjusted Diebold-Mariano test enables us to alleviate the problem through modifying their test statistic. The null hypothesis of the adjusted Diebold-Mariano test is that the benchmark model A is not inferior to any of the alternative model B, i.e., the two models have the same of expected prediction accuracy. Thus, it can be written as follows:

15 $$H_{0} :E[g(e_{t}^{A} ) - g(e_{t}^{B} )] = 0$$

where $e_{t}^{A}$ and $e_{t}^{B}$ denotes the forecast errors of model A and model B, and loss function *g* represents the equality of forecast mean squared errors. Thus, the DM statistics can be defined as:

16 $$ S_{1} = \left[ {\frac{{n + 1 - 2h + n^{ - 1} h(h - 1)}}{n}} \right]\frac{{\mathop d\limits^{\_} }}{{\sqrt {(V(\mathop d\limits^{\_} ))} }}$$

Here

17 $$ \mathop d\limits^{\_} = n^{ - 1} \sum\limits_{t = 1}^{n} {dt} , d_{t} = g(e_{t}^{A} ) - g(e_{t}^{B} ), \, t = 1,...,n$$

and the variance of $\mathop d\limits^{\_}$ is:

18 $$ V(\mathop d\limits^{\_} ) = n^{ - 1} [\gamma_{0} + 2\sum\limits_{k = 1}^{h - 1} {\gamma_{k} } ]$$

Where $\gamma_{k} = n^{ - 1} \sum\limits_{t = k + 1}^{n} {(d_{t} - \mathop d\limits^{\_} } )(d_{t - k} - \mathop d\limits^{\_} )$; $\gamma_{{0}}$ is the variance of $d_{t}$; *h* refers to the h-steps ahead forecasts; and $n$ is the sample size in the testing set.

In addition, we further adopt the approach of model confidence set (MCS) proposed by Hansen et al. (2011) to identify the potential models with superior forecasting ability without a benchmark model to be specified. A MCS is a subset of models that are viewed as the best forecasting models with a certain level of confidence.^5^

Consider a set, ${M}_{0}$, containing a number of models ($i=1,...,m0$). The models are evaluated in terms of a loss function over the sample period,^6^ and the loss associated with model $i$ in period $t$ as ${L}_{i,t}$. Thus, we define the relative performance of different models, ${d}_{ij,t}\equiv {L}_{i,t}- {L}_{j,t} for all i, j \in {M}_{0}$. Then the set of superior models can be defined by:

19 $${M}^{*}\equiv \{i\in {M}_{0}: E({d}_{ij,t}) \le 0\,for\,all\,j\in {M}_{0}\}.$$

The MCS is completed via a sequence of significance tests to trim the set of candidate models, ${M}_{0}$. At each step, the null hypothesis is conducted as follows:

20 $${H}_{0,M}: E({d}_{ij,t}) =0\,for\,all\,i, j\in M \subset {M}_{0}$$

The test aims for the full set of candidate models, $M={M}_{0}$, and if H_0_ is rejected, the worst performing model is eliminated from M. The trimming procedure is repeated until the first non-rejection occurs, and the set of surviving models is the model confidence set (MCS), ${M}^{*}$.^7^ We employ the range statistic and the semi-quadratic statistic for the test as follows:

21 $$T_{R} = \mathop {\max }\limits_{{i,j{ } \in { }M}} \frac{{\left| {\overline{d}_{ij} } \right|}}{{\sqrt {var\left( {d_{ij} } \right)} }},T_{SQ} = \mathop \sum \limits_{i < j} \frac{{\left( {\overline{d}_{ij} } \right)^{2} }}{{var\left( {\overline{d}_{ij} } \right)}}$$

In the paper, we consider the confidence (significance) level of 90% (10%) and compute the MCS p-values based on the range statistics using the circular block bootstrap. If the MCS p-value is less than 0.1, it indicates that the model should be deleted from the set of M_0_, namely, the forecasting ability of the model is weaker than that of other models.

### Data

The European Climate Exchange (ECX) is the carbon trading market with the largest trading volume under the EU ETS, accounting for 80% of the total trading volume in the EU. We collect daily prices of the carbon futures contracts which mature in December 2016 and December 2017, which are denoted as the Dec16 and the Dec17, respectively. For the two futures contracts, the daily data from January 2, 2012 to December 30, 2016 for the Dec16 and January 2, 2013 to 13 October 2017 for the Dec17, without including public holidays, respectively, is obtained from the website of ECX (http://www.theice.com). It results in a total of 1302 and 1241 observations for the two samples, respectively.^8^

For prediction modeling, the sample is divided into the in-sample training set and the out-of-sample testing set, where the training set is used to estimate and optimize the proposed model, and the testing set is used to evaluate the prediction performance of the established model. The detailed division of the two contracts are reported in Table 1. We provide robustness exercises for other forecast sample lengths in Sect. 5. The closing prices of the Dec16 and Dec17 futures contracts are shown in Fig. 15. As shown in the figure, the futures prices illustrate a pattern of time-varying characteristics (e.g., time-varying volatility) and non-normality (e.g., large price movements); in the next section we test the series for nonlinearity as well as non-stationarity.

**Table 1:** Samples of carbon futures prices

Contracts	Sample	Size	Sample period
Dec16	Sample set	1302	2-Jan-2012 ~ 30-Dec-2016
	Training set	1202	2-Jan-2012 ~ 12-Aug-2016
	Testing set	100	12-Aug-2016 ~ 30-Dec-2016
Dec17	Sample set	1241	2-Jan-2013 ~ 13-Oct-2017
	Training set	1141	2-Jan-2013 ~ 26-May-2017
	Testing set	100	29-May-2017 ~ 13-Oct-2017

### Nonstationarity and nonlinearity tests of carbon prices

In this section, we employ the augmented Dicky–Fuller (ADF) and Brock–Decher–Scheikman (BDS) tests to examine the nonstationarity and nonlinearity of Carbon futures prices. In the BDS test, the embedding dimension is usually set to 2–5, and the dimensional distance is set to 0.7 times of the variance of data. The results of the two tests are reported in Tables 2 and 3. As shown in the tables, the ADF test indicates that carbon futures prices are nonstationary at all the levels of significance. Further, the BDS test indicates that the prices are nonlinear at the significance level of 1%. These findings confirm prior results that carbon futures are non-linear, non-stationary series and thus alternative approaches to traditional econometrics are worth investigating.

**Table 2:** ADF test result

		Test critical values:			
		1% level	5% level	10% level	
Contract	t-Statistic	−3.436	−2.864	−2.568	Prob.*
Dec16	−2.288				0.176
Dec17	−2.400				0.142

**Table 3:** BDS test result

Contracts	m-Dimensional space							
	2		3		4		5	
	*t-*Stat	Prob	*t*-Stat	Prob	*t-*Stat	Prob	*t*-Stat	Prob
Dec16	0.032	0.000	0.059	0.000	0.081	0.000	0.093	0.000
Dec17	0.031	0.000	0.060	0.005	0.085	0.000	0.098	0.000

## Empirical results

### EEMD decomposition

**Fig. 3:**
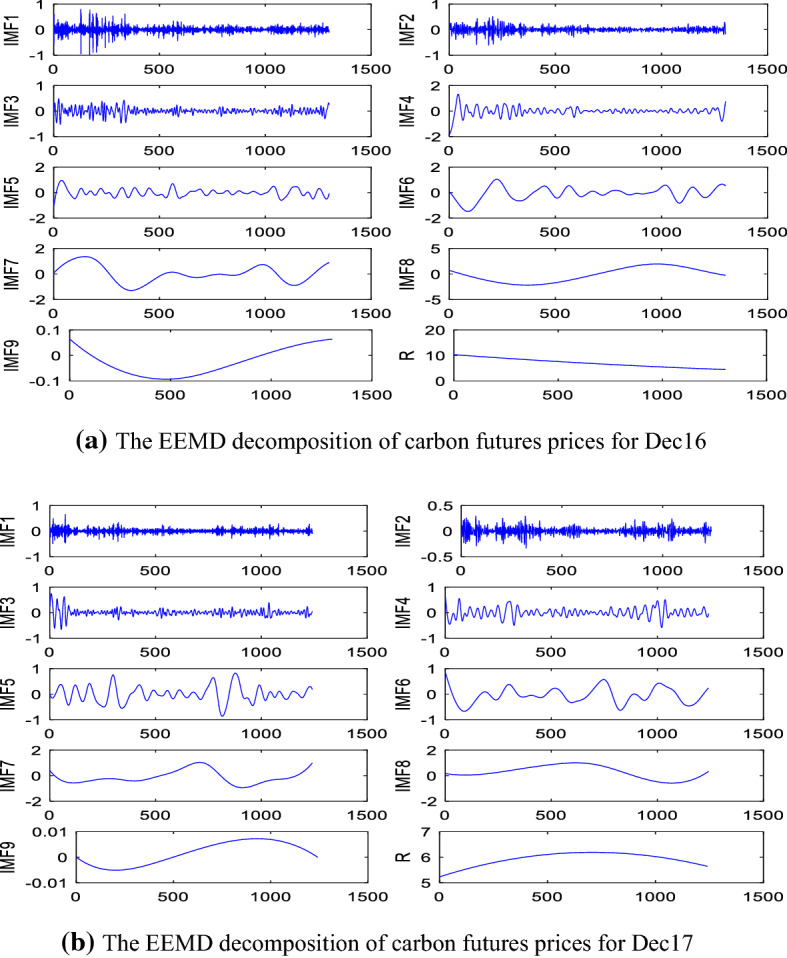
The EEMD decomposition of carbon futures prices

We implement the proposed hybrid model outlined in Sect. 3.4 step by step. First, we apply EEMD method to decompose the carbon prices of the Dec16 and the Dec17. Here the standard deviation of white noise of EEMD decomposition is set to 0.2 times the standard deviation of the carbon futures price, and the number of white noises added is 100. The results of decomposition are reported in Fig. 3.

As shown in Fig. 3, the carbon prices of Dec16 and Dec17 are decomposed into nine IMF functions and one residue (R). The IMFs are arranged by their frequencies, from the highest to the lowest. Since the residue (R) represents the overall trend of the original series, it can be seen from Fig. 3 that the Dec16 contract shows a downward trend during the sample period, while the tendency of Dec17 rises at first and then falls during the entire training period.

### The identification of different frequency components (fuzzy entropy and K-means cluster methods)

**Fig. 4:**
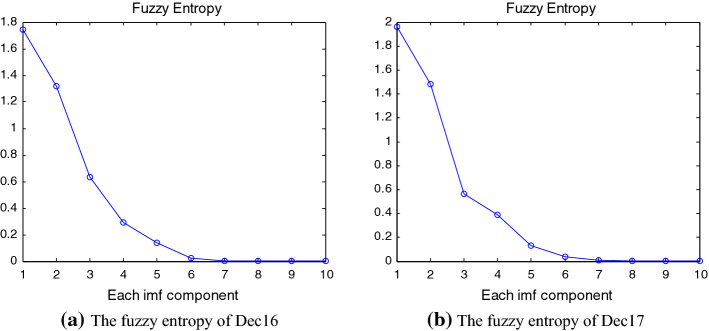
The fuzzy entropy of the IMFs and the residue

After obtaining the decomposed components, we use fuzzy entropy as well as the K-means cluster methods to reconstruct the IMFs and the residue. The fuzzy entropy method is employed to calculate the complexity of the IMFs and the residue, where the embedded dimension *m* = 2, tolerance parameter *r* = $0.2\sigma_{SD}$, and *n* = 2. The fuzzy entropy values of each IMF and the residue of the Dec16 and Dec17 are presented in Fig. 4. As shown in the figure, the fuzzy entropy values of the IMF1 and IMF2 are bigger than 1, while the fuzzy entropy values of the IMF3-IMF9 and the residue are less than 1 for the Dec16. This indicates that the IMF1 and IMF2 have the highest complexity while all other IMFs and the residue have the lower complexity.^9^ In particular, starting from the fuzzy entropy of IMF4, it gradually declines to about 0, indicating the decreasing degree of the complexity. All these show that the high frequency components with the characteristics of higher uncertainty have higher complexity than the low frequency components. Similarly, we find very similar complexity of the different IMFs (residue) of the carbon futures prices for the Dec17.

Since the number of the IMFs and the residue decomposed by the EEMD is large, it takes a lot of observations to adequately train the model as well as long training time for the final modeling if the prediction is directly constructed based on the IMFs. Thus, it is necessary to reconstruct the IMFs and the residue before prediction. In general, high frequency components have the highest fuzzy entropy while low frequency components have minimal fuzzy entropy; the fuzzy entropy values decline monotonically as you move from the first IMF to the second IMF and so on. Since the IMFs with similar frequencies generally have approximate fuzzy entropy values, the fuzzy entropy is a good measure of the similarity for the decomposed IMFs and the residue, which can be used as a clustering factor to reconstruct the decomposed components by the K-means clustering method.

Then, the K-means clustering method is applied to identify different categories for the components of the carbon futures prices for the Dec16 and Dec17 contracts. For the Dec16 contract, the IMF1 and IMF2 are clustered into high-frequency components, IMF3 is clustered into low-frequency components and other components (including the residue) are clustered into trend, respectively. Similarly, for the Dec17 contract, IMF1-IMF2 are clustered into high-frequency components, IMF3-IMF4 are low-frequency components, and other components (including the residue) belong to trend, respectively. Further, the IMFs in each category are aggregated to obtain the high frequency, low frequency and trend series, which reflect the short-term fluctuations (randomness), the strong periodic fluctuations (such as the impact of major events), and the tendency pattern of carbon futures markets, respectively.

**Fig. 5:**
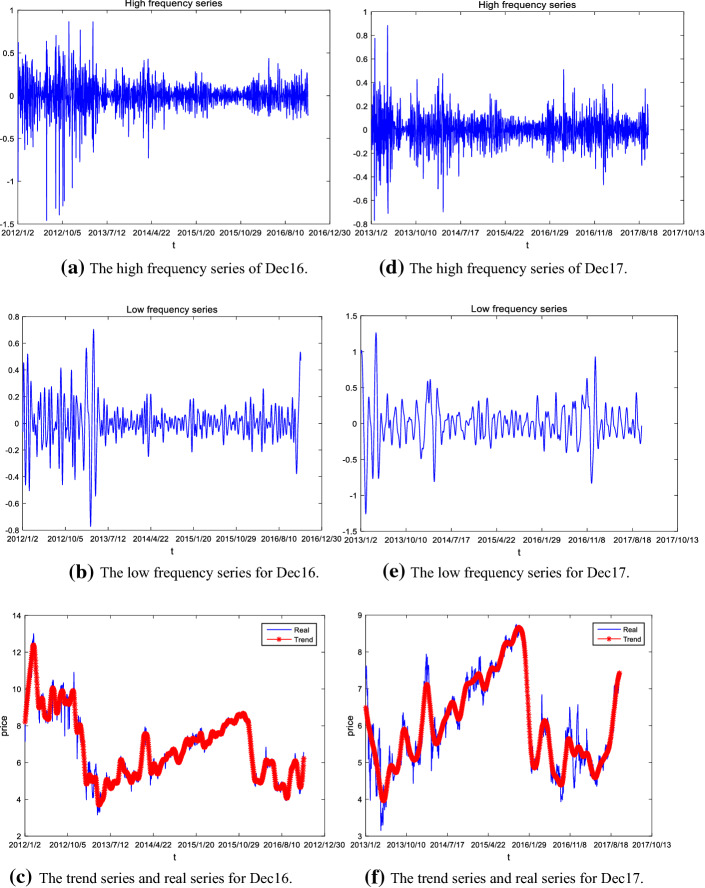
The reconstructed high frequency, low frequency and trend series

The reconstructed high frequency, low frequency and trend series are shown in Fig. 5. For comparisons, the trend series and the real price series are shown in the same figure. As shown in Fig. 5, the trend series accurately depicts the overall trend of the closed prices of carbon futures without losing too much information.

### The identification and modeling of the ARMA model for high and low frequencies series

Under the decomposition-ensemble framework, we first need to further test the stationarity of the three reconstructed series. The results of the Augmented Dickey-Fuller (ADF) test are reported in Table 13 in “Appendix”. As shown in the table, the high and low frequency series of the two contracts are stationary, while the trend series are non-stationary. The high and low frequency series of both contracts are stationary, as anticipated, which supports the use of an ARMA model. However, the trend series of both contracts are non-stationary, also as anticipated, which supports the use of the extreme learning machine (ELM) model to analyze this component.

Using the above reconstructed stationary series, including the high and low frequencies series, to perform the experiments, we employ the ARMA(p, q) model for the prediction. The structure and parameters of the ARMA model are identified by the autocorrelation and partial correlation analysis. Therefore, the optimal ARMA models for the high and low frequency series are identified by the AIC criteria.^10^

#### Out-of-sample prediction of high frequency series

**Fig. 6:**
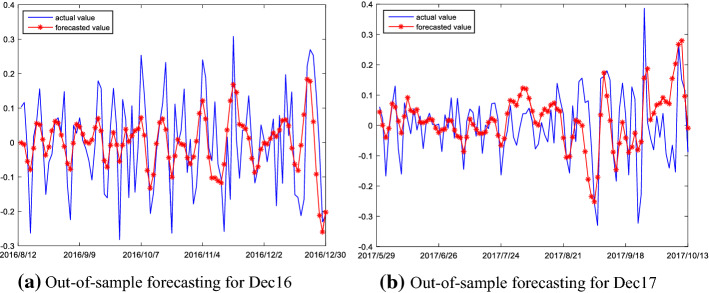
Out-of-sample forecasting result of high frequency series

Finally, the final forecasting results of the high frequency series of the Dec16 and Dec17 are reported in Fig. 6. As shown in the figure, the ARMA model established in this paper can accurately capture the magnitude and direction of the high frequency series changes for the Dec16 and Dec17, and it has strong prediction power and is very suitable for the prediction of high frequency series.

#### Out-of-sample prediction of low frequency series

**Fig. 7:**
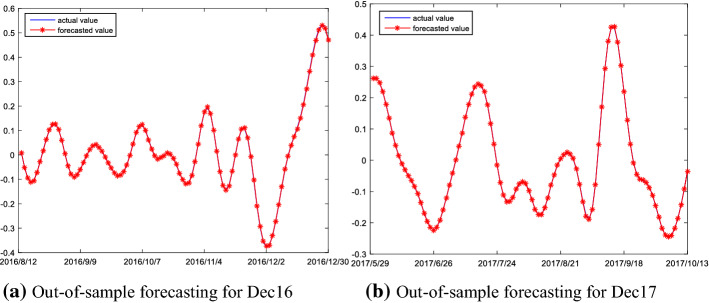
Out-of-sample forecasting result of low frequency series

The results of the prediction of low frequency series of Dec16 and Dec17 are shown in Fig. 7. As shown in the figure, the magnitude and direction of changes are almost completely captured by the ARMA model established in this paper. Thus, the ARMA model has remarkable prediction power on the low frequency series.

### The ELM modeling of trend series

From the results of stability test in the above section, we find that the trend component is non-stationary. Thus, this section employs the ELM method for modeling the trend series because of its strong prediction power in other contexts for time-series which are nonlinear and non-stationary. Before training the ELM model, it is necessary to identify the number and the activation function of neurons in the hidden layer. Therefore, we divide the training sample into a training set and a test set, and then use time-varying cross-validation and grid search methods to identify the optimal number and the optimal activation function of neurons in the hidden layer.^11^

**Fig. 8:**
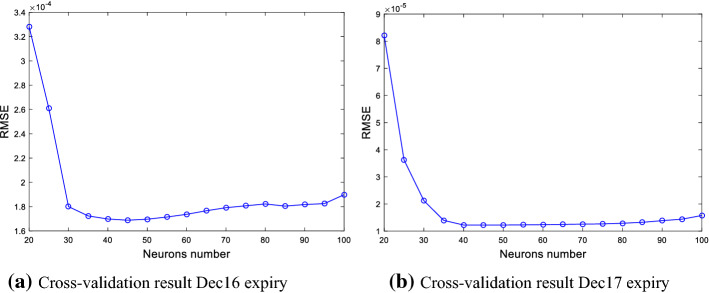
The RMSE of the cross-validation and grid search methods

The results of the time-varying cross-validation and grid search methods are reported in Fig. 8. For the trend series of the Dec16 and the Dec17, the root mean square error (RMSE) of the cross-validation monotonically decreases when the number of neurons in the hidden layer is in Liu and Shi (2013); Zhu & Wei, 2013), while the RMSE basically remains the same or increases slightly as the number of neurons increases during the interval of [45,100]. It indicates that the RMSE of the cross-validation method is smallest when the number of neurons in the hidden layer is around 45. Therefore, the number of neurons in the ELM model is set to 45 for the trend series of the two contracts.

**Fig. 9:**
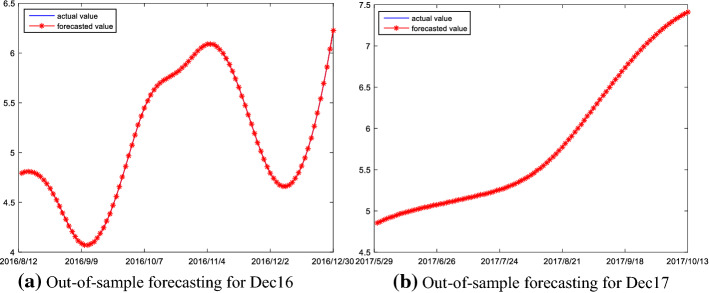
Out-of-sample forecasting result of trend frequency series

The forecasting results of the Dec16 and the Dec17 are shown in Fig. 9; the figure shows the curves of out-of-sample forecast are almost coincident with their real curves. This indicates that the change magnitudes and movement directions of the trend series are remarkably well captured by the ELM model. This is mainly attributed to the following: on the one hand, the original prices are decomposed into high-frequency, low-frequency and trend components via using the EMMD method, which may solve the problem of mixed frequency components in the prices; on the other hand, when using the reconstruction algorithm proposed in this paper, the decomposed components of carbon prices is reconstructed into three different frequency series according to the complexity of the components. This reconstruction algorithm is advantageous in that it makes the structure of the high/low frequency and trend series simpler, which facilitates the ELM modeling. Thus, all this indicates that the ELM model is highly suitable for the prediction of trend series component.

### Forecasting results of the proposed hybrid model

#### Comparison between the proposed hybrid model and other models

We aggregate the forecasting results of the three different frequency series (including high frequency, low frequency and trend series) for the Dec16 and the Dec17 and generate the ensemble forecasting result based on the proposed EEMD-FuzzyEn–ARMA-ELM hybrid model. For comparisons, we employ the single ARMA and ELM models, Random walk with drift item, multiscale ensemble prediction models, including the EEMD-FuzzyEn-ARMA and EEMD-FuzzyEn-ELM models, to predict carbon prices as well. The evaluation of out-of-sample forecasting results for the two carbon futures contracts is reported in Table 4.

**Table 4:** Comparison of out-of-sample forecasting for different models

Contract	Error	ARMA	ELM	RW	EEMD-FuzzyEn-ARMA	EEMD-FuzzyEn-ELM	EEMD- FuzzyEn-ARMA -ELM
Dec16	MAPE (%)	3.108	3.113	3.030	**1.997**	**1.776**	**1.661**
	RMSE	0.1995	0.1941	0.1890	**0.1318**	**0.1156**	**0.1054**
	R	0.9254	0.9295	0.9323	**0.9674**	**0.9755**	**0.9798**
	DS	0.5960	0.5857	0.5758	**0.8182**	**0.6869**	**0.8182**
Dec17	MAPE (%)	1.735	1.724	1.700	**1.232**	**1.174**	**1.220**
	RMSE	0.1497	0.1496	0.1491	**0.1004**	**0.0929**	**0.0979**
	R	0.9675	0.9681	0.9677	**0.9857**	**0.9872**	**0.9863**
	DS	0.5152	0.5354	0.5455	**0.7172**	**0.6869**	**0.7172**

Here RW refers to Random walk with drift item. In the bold cells, the number in each cell is the statistics of measuring forecast error for the proposed hybrid models in the paper

Table 4, in general, shows that the prediction performance of the proposed EEMD-FuzzyEn-ARMA-ELM hybrid model is very good relative to that of the other models considered for both the Dec16 and the Dec17. In term of the forecast accuracy, documented by the MAPE and RMSE, all the multiscale ensemble prediction models perform much better than the single prediction models (e.g. ARMA and RW). This indicates that the hybrid forecasting models based on the decomposition-reconstruction principle have strong prediction power and substantially improves the prediction accuracy compared to the single models. Furthermore, among the ensemble prediction models, the prediction accuracy of the hybrid models incorporating ELM is superior over that of the hybrid model incorporating ARMA; thus the novel incorporation of ELM into the model is very beneficial. This is mainly attributed to the advantage of ELM in forecasting non-linear and non-stationary series, while the ARMA model is useful for traditional linear, stationary series it is not designed to capture non-linearities or non-stationarities.

The correlation coefficient (R) for the hybrid models is higher than the single ARMA model, the ELM model or random walk model with drift term. The higher correlation coefficient represents the higher degree of association between the predicted and actual values, indicating the hybrid models greatly improve the forecasting accuracy. In terms of the direction prediction, the direction statistics DS, we find that the proposed hybrid models achieve the highest rates of directional accuracy. The hybrid models perform much better than the simple models and rates of 70–80% are impressive given we are looking at noisy daily data. In addition, the ARMA model outperforms the ELM model in capturing the direction prediction of carbon price changes, either in the single model or in the ensemble models.

Following Harvey et al. (1997), we use the adjusted Diebold-Mariano (DM) test to assess the differences between the performance of two forecasts in the paper. The results of the comparison of the forecasting performance for the two contracts are reported in Table 5. We can draw the following conclusions. First, the prediction accuracy of the ensemble models (EEMD-FuzzyEn-ARMA, EEMD-FuzzyEn-ELM and EEMD-FuzzyEn-ARMA-ELM) is significantly better than that of the single models (ARMA, ELM, RW). Second, the proposed hybrid EEMD-FuzzyEn-ARMA-ELM model, in general, demonstrates the superior performance of carbon price prediction than other proposed ensemble models and all the single models. However, for the Dec17 contract statistically there is not a difference between the prediction performance of EEMD-FuzzyEn-ARMA-ELM and EEMD-FuzzyEn-ELM; nevertheless, this underlines that models that feature EEMD, Fuzzy Entropy and Extreme Learning Machine (as both these do) are highly suitable for this application.

**Table 5:** The results of the adjusted DM test for different forecasting models

	Alternative model	Benchmark model					
		ARMA	ELM	RW	EEMD-FuzzyEn-ARMA	EEMD-FuzzyEn-ELM	EEMD-FuzzyEn-ARMA-ELM
Dec16	ELM	−0.8825 (0.1898)					
	Rw	−1.972** **(0.0257)**	−1.106 (0.1357)				
	EEMD-FuzzyEn-ARMA	−3.983*** **(0.0000)**	−3.628*** **(0.0000)**	−3.647*** **(0.0000)**			
	EEMD-FuzzyEn-ELM	−4.794*** **(0.0000)**	−4.710 *** **(0.0000)**	−4.800*** **(0.0000)**	−2.087** **(0.0197)**		
	EEMD-FuzzyEn-ARMA-ELM	−4.993*** **(0.0000)**	**−4.898** **(0.0000)**	−5.008*** **(0.0000)**	−3.336*** **(0.0000)**	−2.728*** **(0.0038)**	
**Dec17**	ELM	−0.0468 (0.4814)					
	Rw	−0.3181 (0.3756)	−0.1359 (0.4461)				
	EEMD-FuzzyEn-ARMA	−2.106** **(0.0189)**	−2.1916** **(0.015)**	−2.103** **(0.0190)**			
	EEMD-FuzzyEn-ELM	−2.508*** **(0.0069)**	−2.6239*** **(0.0050)**	−2.512*** **(0.0068)**	−1.080 (0.1414)		
	EEMD-FuzzyEn-ARMA-ELM	−2.209** **(0.0148)**	−2.301** **(0.0118)**	−2.208** **(0.0148)**	−2.646*** **(0.0047)**	0.7711 (0.2212)	

Test of Diebold-Mariano (DM) examines the null hypothesis that there is no difference of the forecasting performance between the benchmark model and the alternative model. *** indicates *p* < .01; ** indicates < .05; and * indicates *p* < .10. The value in parentheses is *P* value. RW refers to the Random walk with drift item

In addition, we employ the method of model confidence set (i.e., MCS) proposed by Hansen et al. (2011) to examine the potential models with superior forecasting ability. To obtain the statistics of TR and TSQ and their corresponding MCS p-values, we consider the confidence level of 90% (i.e., significance level $\alpha =0.10$) for the MCS as suggested by Hansen et al. (2011). The block length is set to 3, and the number of bootstrap samples is 10,000. Table 6 reports the empirical results of the MCS test of the forecasting performance for forecasting models. As shown in the table, under different loss functions ((i.e., *MSE, MAE, MAPE,* and *QLIKE*), only hybrid models ever survive in the model confidence set. In particular, the proposed hybrid method of EEMD-FuzzyEn-ARMA-ELM consistently enters the MCS for the Dec 16 and Dec 17.^12^ This indicates that our proposed hybrid model generates forecasts that are statistically better than it, which is generally consistent the DM test findings (Table 5).

**Table 6:** The MCS p-value of different models for forecasting carbon futures prices

Contract	Model	MSE		MAE		MAPE		QLIKE	
		T_R_	T_SQ_	T_R_	T_SQ_	T_R_	T_SQ_	T_R_	T_SQ_
**Dec16**	M1	0.0000	0.0000	0.0000	0.0000	0.0000	0.0000	0.0000	0.0000
	M2	0.0000	0.0000	0.0000	0.0000	0.0000	0.0000	0.0000	0.0000
	M3	0.0000	0.0000	0.0000	0.0000	0.0000	0.0000	0.0000	0.0000
	M4	0.0122	0.0039	0.0200	0.0120	0.0214	0.0117	0.0065	0.0021
	M5	0.0122	0.0052	0.0669	0.0669	0.0532	0.0532	0.0065	0.0037
	M6	**1.0000**	**1.0000**	**1.0000**	**1.0000**	**1.0000**	**1.0000**	**1.0000**	**1.0000**
**Dec17**	M1	0.0442	0.0290	0.0035	0.0060	0.0008	0.0045	0.0228	0.0145
	M2	0.0438	0.0226	0.0028	0.0053	0.0008	0.0037	0.0206	0.0126
	M3	0.0442	0.0270	0.0040	0.0088	0.0017	0.0062	0.0228	0.0137
	M4	**0.1392**	**0.2210**	**0.6397**	**0.5781**	**0.7353**	**0.6479**	**0.2715**	**0.2893**
	M5	**1.0000**	**1.0000**	**1.0000**	**1.0000**	**1.0000**	**1.0000**	**1.0000**	**0.4606**
	M6	**0.4925**	**0.4925**	**0.6397**	**0.5781**	**0.7353**	**0.6479**	**0.4606**	**1.0000**

The six forecasting models (referred to as M1-M6) are ARMA, ELM, RW, EEMD-FuzzyEn-ARMA, EEMD-FuzzyEn-ELM, EEMD-FuzzyEn-ARMA-ELM, respectively. The numbers in the cells are the p-values corresponding to the MCS test using 10,000 Bootstrap simulations. The p-value is marked in bold and underlined when it is greater than 0.10, indicating the survival model during the MCS test achieves a better predictive ability

**Fig. 10:**
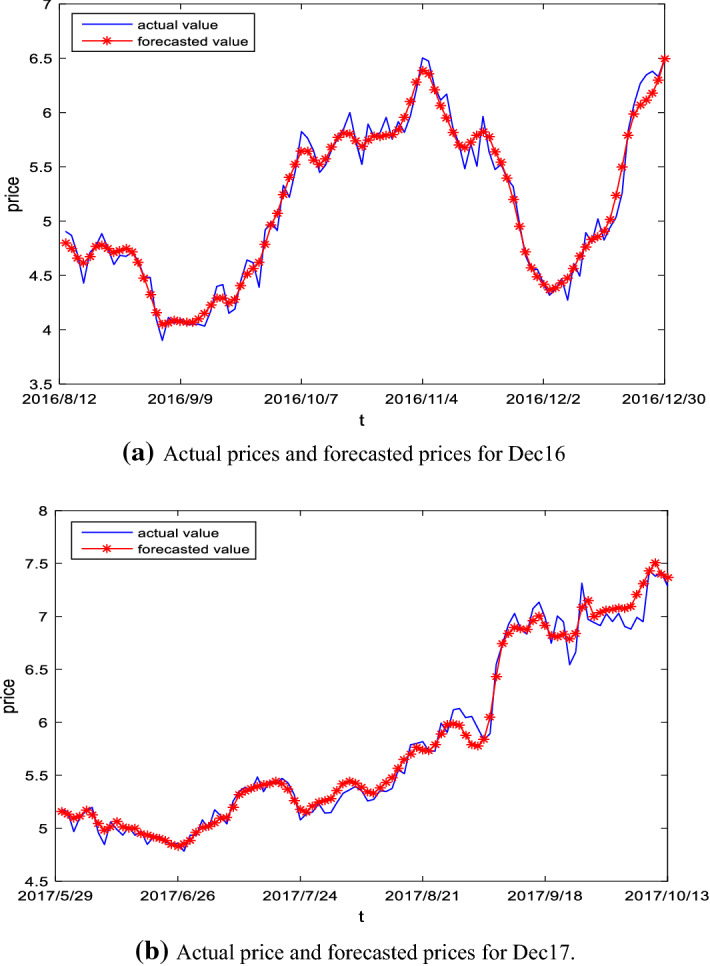
Actual price and forecasting prices for Dec16 and Dec17

Figure 10 demonstrates the out-of-sample prediction results of carbon futures prices for the Dec16 and Dec17 using the proposed forecasting model. As shown in the figure, the proposed hybrid model has a strong power in the forecasting of carbon future prices, since it can not only accurately measure the magnitude of carbon futures price changes, but also capture the future direction information in price changes.

#### Comparison of prediction performance of the proposed models between different reconstruction algorithms (FuzzyEn-K-mean and fine-to-coarse)

In order to examine the prediction power of the proposed reconstruction algorithms, we further compare the differences of prediction performance for the hybrid models under different reconstruction algorithms, between the proposed reconstruction algorithm and the classical fine-to-coarse reconstruction algorithm.

Table 7 reports the out-of-sample prediction result of the proposed hybrid models using different reconstruction algorithms, including the proposed FuzzyEn-K-mean and traditional fine-to-coarse algorithms. As shown in the table, compared to the fine-to-coarse algorithm, the proposed Fuzzy Entropy and K-mean clustering method is superior. The results show that with the same conditions, the performance of the hybrid models incorporating the proposed algorithm (including EEMD-FuzzyEn-ARMA, EEMD-FuzzyEn-ELM, and EEMD-FuzzyEn-ARMA) is much better than that of the models incorporating fine-to-coarse algorithm. For the Dec16, this reduces the horizontal prediction error index (MAPE) by 38.72%, 45.70% and 49.03%, respectively, and reduces the RMSE error index by 34.49%, 44.64% and 47.61%, respectively. Further, in term of the direction statistics DS index, the hybrid model under the proposed reconstruction algorithm can achieve a higher prediction accuracy by 49.99%, 28.30% and 49.99%, respectively. The prediction results for the Dec17 is very similar to the case of the Dec16, which confirms the robustness of the strong prediction performance of the hybrid model under proposed algorithm in our paper.

**Table 7:** Comparison of prediction performance for the hybrid models under different reconstruction algorithms

Contract	Error	EEMD-FTC-ARMA	EEMD-FTC-ELM	EEMD-FTC-ARMA-ELM	EEMD-FuzzyEn-ARMA	EEMD-FuzzyEn-ELM	EEMD-FuzzyEn-ARMA-ELM
Dec16	MAPE (%)	3.259	3.271	3.259	**1.997** **(38.72%)**	**1.776** **(45.70%)**	**1.661 (49.03%)**
	RMSE	0.2012	0.2088	0.2012	**0.1318 (34.49%)**	**0.1156 (44.64%)**	**0.1054 (47.61%)**
	R	0.9305	0.9213	0.9305	**0.9674** **(3.97%)**	**0.9755** **(4.84%)**	**0.9798 (5.30%)**
	DS	0.5455	0.5354	0.5455	**0.8182 (49.99%)**	**0.6869** **(28.30%)**	**0.8182 (49.99%)**
Dec17	MAPE (%)	1.662	1.627	1.662	**1.232** **(25.87%)**	**1.174** **(27.84%)**	**1.220** **(26.59%)**
	RMSE	0.1412	0.1391	0.1412	**0.1004** **(28.90%)**	**0.0929** **(33.21%)**	**0.0979 (30.67%)**
	R	0.9704	0.9713	0.9704	**0.9857** **(1.48%)**	**0.9872** **(1.64%)**	**0.9863** **(1.64%)**
	Ds	0.5051	0.5354	0.5051	**0.7172** **(41.99%)**	**0.6869** **(28.30%)**	**0.7172 (41.99%)**

Here FTC refers to the fine-to-coarse reconstruction algorithm. **In the bold cells**, the first number in each cell is the statistics of measuring forecast error for the proposed hybrid models in the paper, and the value in parentheses is the percent of the increase in prediction accuracy using the same model under different reconstruction algorithms between proposed reconstruction and fine-to-coarse

**Fig. 11:**
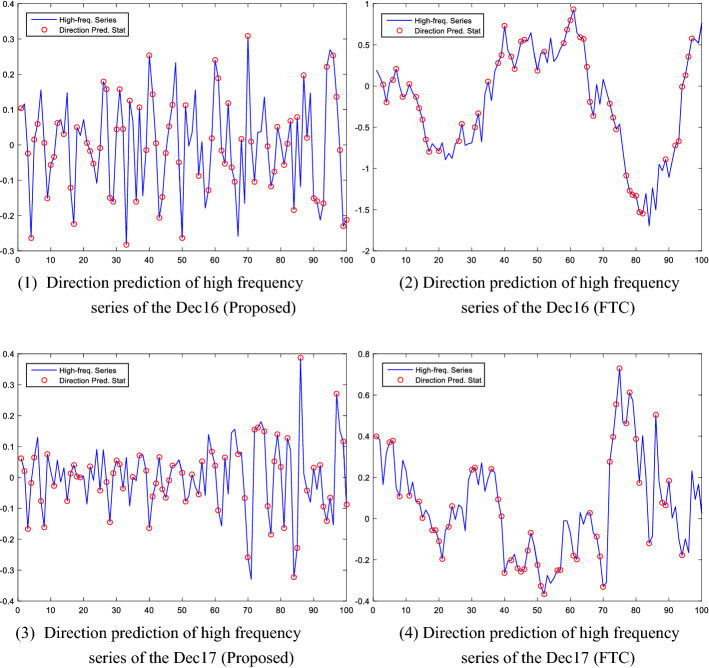
The direction prediction of high frequency series under different reconstruction algorithms Notes: “Proposed” refers to the proposed reconstruction algorithm, using the Fuzzy Entropy and K-means clustering methods in the paper; "FTC" represents the traditional fine-to-coarse reconstruction algorithm

Further, we report the direction prediction results of the high-frequency series under two different reconstruction algorithms in Fig. 11. As shown in the figure (as well as in Table 5), we find that the hybrid model under the proposed reconstruction algorithm (using the fuzzy entropy and K-means clustering methods) outperforms the model under the traditional fine-to-coarse algorithm in capturing the prediction of direction movements in the carbon prices of both the Dec16 and Dec17. For example, the proposed hybrid EEMD-FuzzyEn-ARMA-ELM model accurately predicts the 75 direction changes of high-frequency series among 100 predicted observations for the Dec16, while EEMD-FTC-ARMA-ELM only accurately predicts 51 direction changes among the 100 observations. Similarly, the hybrid model under the proposed reconstruction algorithm demonstrates a higher power of direction prediction than the model under the traditional fine-to-coarse algorithm for the Dec17.

In order to compare the forecasting performance of the hybrid models with the proposed FuzzyEn-K-mean to the traditional fine-to-coarse algorithms, we use the adjusted Diebold-Mariano test (Harvey et al., 1997) to assess their predictive performance. The results of tests of equal predictive performance between two reconstruction algorithms for the Dec16 and Dec17 are reported in Table 8. We can draw the following conclusions. Overall, with the same conditions, the prediction accuracy of the ensemble models based on the proposed reconstruction algorithm (using the Fuzzy entropy and K-means clustering methods), including EEMD-FuzzyEn-ARMA, EEMD-FuzzyEn-ELM and EEMD-FuzzyEn-ARMA-ELM, is significantly better than that of the ensemble models based on the traditional fine-to-coarse algorithm.

**Table 8:** The results of the adjusted DM test for the hybrid models under different reconstruction algorithms

Contract	Alternative model	Benchmark model		
		EEMD-FTC-ARMA	EEMD-FTC-ELM	EEMD-FTC-ARMA-ELM
Dec16	EEMD-FuzzyEn-ARMA	−4.136*** (0.000)	−3.878*** (0.000)	−4.137*** (0.000)
	EEMD-FuzzyEn-ELM	−5.386*** (0.000)	−4.883*** (0.000)	−5.387*** (0.000)
	EEMD-FuzzyEn-ARMA-ELM	−5.707*** (0.000)	−5.147*** (0.000)	−5.707*** (0.000)
Dec17	EEMD-FuzzyEn-ARMA	−1.980** (0.025)	−1.994** (0.025)	−1.979** (0.025)
	EEMD-FuzzyEn-ELM	−2.458*** (0.008)	−2.495*** (0.007)	−2.458*** (0.008)
	EEMD-FuzzyEn-ARMA-ELM	−2.102** (0.019)	−2.123** (0.018)	−2.208** (0.015)

*Test* of Diebold-Mariano (DM) examines the null hypothesis that there is no difference of the forecasting performance between the benchmark model and the alternative model. “FuzzyEn” refer to the proposed reconstruction algorithm of Fuzzy entropy and K-means clustering methods; "FTC" represents the traditional fine-to-coarse reconstruction algorithm. *** indicates *p* < .01; ** indicates < .05; and * indicates *p* < .10. The value in parentheses is *P* value

In sum, our evidence clearly supports the proposed hybrid model which demonstrates superior performance in carbon price prediction than other ensemble models and all the single models. Compared to the fine-to-coarse algorithm, the proposed Fuzzy Entropy and K-mean clustering methods has the advantage that they can easily analyze the complexity of the IMFs and the residue and thus reconstruct the three different frequency series more efficiently and accurately for prediction. Further, we benefit from the advantages of the prediction power of the ARMA model for the stationary high and low frequency series and the extreme learning machine (ELM) method for the non-stationary trend series. The forecasts of all three components (high frequency, low frequency and trend) based on these forecasting models are aggregated to form the final forecast of the carbon price. Therefore, the new hybrid forecasting model under the decomposition-reconstruction principle proposed in this paper significantly improves the prediction accuracy of carbon futures prices.

## Further analysis

### Robustness tests

In this section, for robustness testing of the prediction ability of the proposed hybrid models, we proceed a couple of out-of-sample predictions using alternative testing samples for the Dec16, including the size of testing samples with 200, 300, 400 and 500 observations, respectively. This means that we reserve the samples of 200, 300, 400, and 500 as the testing sets, while the other observations of the Dec16 are used to train the proposed models. we follow the same steps used in the above section to examine the prediction performance for different models.

Table 9 reports the comparison of the forecasting results of these four different periods. We find that the results of robustness tests of the prediction performance of the hybrid models are consistent with the previous findings. First, the results show that the hybrid models outperform the single models in the prediction of the price level. For example, when using the testing set of 200 observations, compared with the single ARMA model, the ELM model and the random walk model with drift term, the proposed hybrid models increases the prediction accuracy by more than 44%, on average than these single models. Similar, we find the very same results of prediction when using the testing set of 300, 400 or 500 observations. Second, in terms of the diction prediction of price movements, the direction statistics DS is more than 70% for the proposed hybrid models, while only about 50% for the single models. In particular, the hybrid models incorporating the ARMA model, including EEMD-FuzzyEn-ARMA-ELM and EEMD-FuzzyEn-ARMA models, achieve a high level of prediction accuracy in the direction movements at about 77% and 76% for the testing sample of 200 observations of the Dec16.

**Table 9:** The forecasting results for the Dec16 using different testing samples

Size	Error	ARMA	ELM	RW	EEMD-FuzzyEn-ARMA	EEMD-FuzzyEn-ELM	EEMD- FuzzyEn- ARMA- -ELM
200 Obs	MAPE(%)	2.740	2.754	2.688	**1.783**	**1.568**	**1.510**
	RMSE	0.1902	0.1903	0.1817	**0.1209**	**0.1082**	**0.1029**
	R	0.9246	0.9243	0.9314	**0.9698**	**0.9757**	**0.9781**
	Ds	0.5226	0.5075	0.5176	**0.7739**	**0.7337**	**0.7588**
300 Obs	MAPE(%)	2.459	2.525	2.458	**1.425**	**1.405**	**1.324**
	RMSE	0.1813	0.1857	0.1764	**0.1037**	**0.1025**	**0.0969**
	R	0.9811	0.9801	0.9823	**0.9938**	**0.9939**	**0.9945**
	Ds	0.5017	0.4883	0.5084	**0.7291**	**0.7592**	**0.7358**
400 Obs	MAPE(%)	2.068	2.070	2.054	**1.265**	**1.210**	**1.139**
	RMSE	0.1645	0.1614	0.1590	**0.0983**	**0.0939**	**0.0884**
	R	0.9882	0.9887	0.9892	**0.9958**	**0.9962**	**0.9966**
	Ds	0.4987	0.4937	0.4987	**0.7118**	**0.6842**	**0.7118**
500 Obs	MAPE(%)	1.951	1.958	1.928	**1.250**	**1.178**	**1.082**
	RMSE	0.1611	0.1596	0.1564	**0.0999**	**0.0947**	**0.0866**
	R	0.9870	0.9872	0.9878	**0.9950**	**0.9955**	**0.9962**
	Ds	0.4850	0.4810	0.4990	**0.7114**	**0.6814**	**0.7094**

**Fig. 12:**
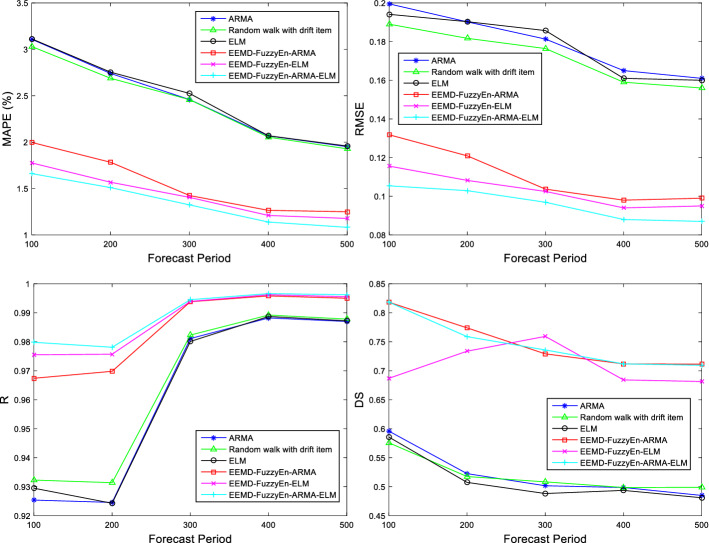
The forecasting results of different models across different testing sample sizes

Here RW refers to Random walk with drift item. In the bold cells, the number in each cell is the statistics of measuring forecast error for the proposed hybrid models in the paper,

Figure 12 presents the forecasting results of different models using different testing sample periods. As shown in the figure, in terms of the level prediction, the indicators of the MAPE, the RMSE and the correlation coefficients (R) of the hybrid forecasting model (i.e., EEMD-FuzzyEn-ARMA-ELM) proposed in this paper are significantly better than those of all other forecasting models under different periods. Second, the proposed hybrid models outperform the single models in the diction prediction of price movements. Although the EEMD-FuzzyEn-ARMA-ELM model is not always superior to other hybrid models in terms of price direction prediction, its forecast performance still ranks the second in all forecasting models. The high DS statistics of the proposed hybrid model, which exceeds 70% in all five forecasting periods, demonstrates high ability to capture the direction of price movements within different forecasting periods.

### The COVID-19 pandemic period

**Fig. 13:**
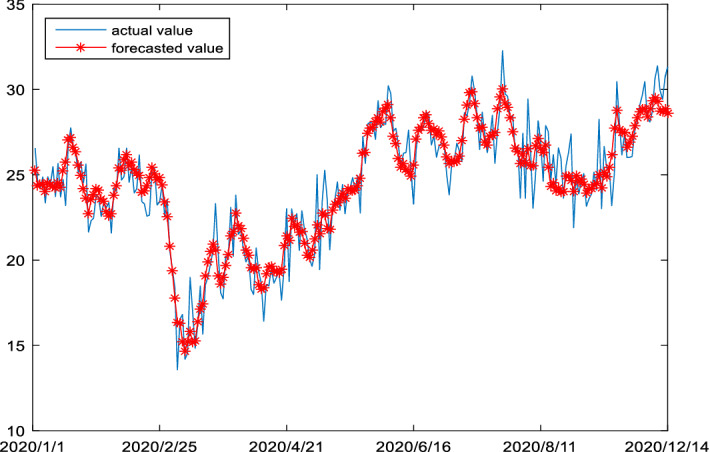
Actual price and forecasting prices for Dec20

**Fig. 15:**
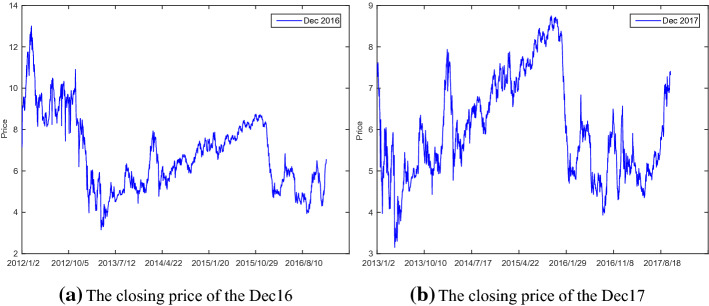
The closing price of the Dec16 and Dec17

The COVID-19 pandemic has a substantial impact on financial markets, including the carbon futures market as well as the economies of many countries during 2020. How reliable is the forecasting performance of the hybrid proposed model during this period on pandemic and great uncertainty? We therefore follow the same framework to examine the extreme data during the pandemic period.^13^ Figure 13 presents the out-of-sample prediction result of carbon futures prices for the Dec20 using the proposed hybrid forecasting model. As shown in the figure, the result demonstrates that our proposed hybrid model has a strong power in the forecasting of carbon futures prices during the pandemic period, capturing the magnitude of carbon futures price changes as well as the direction information in price dynamics well. In fact, this should not be surprising since the hybrid model is well suited to capture the effects of high uncertainty witnessed during the COVID pandemic period.

The result of out-of-sample forecasting performance for the carbon futures contracts of the Dec20 are reported in Table 10. As shown in the table, in general, it indicates that the prediction performance of the proposed EEMD-FuzzyEn-ARMA-ELM hybrid model is very good relative to that of other models. In term of the forecast accuracy, documented by the MAPE and RMSE, all the multiscale ensemble prediction models perform much better than the single prediction models (e.g. ARMA, ELM, and RW).

**Table 10:** Comparison of out-of-sample forecasting performance for the Dec20

Contract	Error	ARMA	ELM	RW	EEMD-FuzzyEn-ARMA	EEMD-FuzzyEn-ELM	EEMD- FuzzyEn-ARMA -ELM
Dec20	MAPE (%)	5.1043	5.4555	5.0823	**4.8577**	**4.1012**	**3.9311**
	RMSE	1.5330	1.6031	1.5315	**1.4666**	**1.2239**	**1.1776**
	R	0.8220	0.8053	0.8223	**0.8381**	**0.8869**	**0.8952**
	DS	0.5060	0.4940	0.5060	**0.7390**	**0.6707**	**0.7390**

Here RW refers to Random walk with drift item. In the bold cells, the number in each cell is the statistics of measuring forecast error for the proposed hybrid models in the paper

This further indicates that the hybrid forecasting models based on the decomposition-reconstruction principle have strong prediction power and substantially improves the prediction accuracy even during the pandemic period. Among the ensemble prediction models, the prediction accuracy of the hybrid models incorporating both ARMA and ELM is superior over that of the hybrid model incorporating only ARMA or ELM. Furthermore, in terms of the correlation coefficient (R), the higher coefficient indicates that our proposed hybrid model outperforms other models in forecasting accuracy. Lastly, based on the direction prediction, the direction statistics (DS), we find that the proposed hybrid model achieve the highest rates of directional accuracy. In addition, even considering the sample of the pandemic period, the hybrid models generally perform much better than the simple models.We further use the adjusted Diebold-Mariano (DM) test to compare the forecasting performance across different models for the Dec20 contract. As shown in Table 11, it indicates the prediction accuracy of the ensemble models incorporating the Extreme Learning Machine (i.e., ELM) is significantly better than that of the single models. Second, the proposed hybrid EEMD-FuzzyEn-ARMA-ELM model, demonstrates superior forecasting performance for carbon price than to all other proposed models.

**Table 11:** Results of the adjusted DM test of different forecasting models for Dec20

	Alternative model	Benchmark model					
		ARMA	ELM	RW	EEMD-FuzzyEn-ARMA	EEMD-FuzzyEn-ELM	EEMD-FuzzyEn-ARMA-ELM
**Dec20**	ELM	2.0828 (0.019)					
	RW	−0.2625 (0.397)	−2.218** (0.014)				
	EEMD-FuzzyEn-ARMA	**-0.899** **(0.185)**	−1.800** **(0.0365)**	**−0.877** **(0.1906)**			
	EEMD-FuzzyEn-ELM	−4.702*** **(0.0000)**	−5.017*** **(0.0000)**	−4.664*** **(0.0000)**	−4.0687*** **(0.0000)**		
	EEMD-FuzzyEn-ARMA-ELM	−4.920*** **(0.0000)**	−5.202*** **(0.0000)**	−4.873*** **(0.0000)**	−5.1563*** **(0.0000)**	−1.936** **(0.0270)**	

Test of Diebold-Mariano (DM) examines the null hypothesis that there is no difference of the forecasting performance between the benchmark model and the alternative model. *** indicates *p* < .01; ** indicates < .05; and * indicates *p* < .10. The value in parentheses is *P* value. RW refers to the Random walk with drift item

Finally, we employ the MCS test to examine the potential group of models with superior forecasting ability for the Dec20. Table 12 reports the empirical results of the MCS test of the forecasting performance for different models. As shown in the table, under different loss functions, the proposed hybrid method of EEMD-FuzzyEn-ARMA-ELM is consistently the only model in the MCS. This is broadly consistent with the empirical findings of the MCS test for the Dec16 and Dec17. This further emphasizes the importance of incorporating Fuzzy Entropy and Extreme Learning Machine when forecasting the carbon price.

**Table 12:** MCS *p*-value of forecasting models for Dec20carbon futures prices

Contract	Model	MSE		MAE		MAPE		QLIKE	
		T_R_	T_SQ_	T_R_	T_SQ_	T_R_	T_SQ_	T_R_	T_SQ_
Dec20	M1	0.0003	0.0000	0.0000	0.0001	0.0006	0.0000	0.0002	0.0001
	M2	0.0003	0.0000	0.0001	0.0001	0.0006	0.0000	0.0002	0.0001
	M3	0.0003	0.0001	0.0001	0.0001	0.0006	0.0000	0.0002	0.0001
	M4	0.0011	0.0014	0.0009	0.0010	0.0035	0.0025	0.0051	0.0026
	M5	0.0458	0.0458	0.0563	0.0563	0.0402	0.0402	0.0251	0.0251
	M6	**1.0000**	**1.0000**	**1.0000**	**1.0000**	**1.0000**	**1.0000**	**1.0000**	**1.0000**

*Notes* The six forecasting models (referred to as M1-M6) are ARMA, ELM, RW, EEMD-FuzzyEn-ARMA, EEMD-FuzzyEn-ELM, EEMD-FuzzyEn-ARMA-ELM, respectively. The numbers in the cells are the p-values corresponding to the MCS test using 10,000 Bootstrap simulations. The p-value is marked in bold and underlined when it is greater than 0.10, indicating the survival model during the MCS test achieves a better predictive ability

**Table 13:** The results of ADF test

		Test critical values				
		1% level	5% level	10% level		
Contract	Reconstructed Series	t-Statistic	−3.436	−2.864	−2.568	Prob.*
Dec16	High	−20.82				0.0000
	Low	−12.56				0.0000
	Trend	−2.230				0.1958
Dec17	High	−18.98				0.0000
	Low	−9.074				0.0000
	Trend	−1.9416				0.8769

Table 13 presents the result of the stationarity test (ADF) of the three reconstructed series (high frequency, low frequency and trend series) of the Dec16 and the Dec17 contracts. The result indicates that the high and low frequency series of the two contracts are stationary, while the trend series of the contracts are non-stationary

In summary, the empirical evidence provided indicates that the forecasting results obtained by the hybrid prediction model based on EEMD decomposition and reconstruction are significantly better than the single prediction models across different testing sample periods as well as the COVID-19 pandemic period, which indicates the proposed framework proposed in this paper is reasonable and robust. The hybrid prediction model using the decomposition-reconstruction algorithm consistently achieves more accurate prediction results both in terms of predicting the magnitude of carbon futures price change as well as the direction carbon futures price movements.

## Conclusions

In this paper, we propose a novel hybrid model that builds on ensemble empirical mode decomposition (EEMD) models by incorporating fuzzy entropy (*FuzzyEn*) and extreme learning machine (ELM) methods. We successfully apply this method to forecast EU carbon futures prices that are characterized by complexity and chaos.

First, we find strong forecasting performance of the EEMD model that includes FuzzyEn and ELM both in terms of predictive accuracy and in terms of sign prediction. This model also performs substantially better than simpler models such as ARMA and random walk; for predictive accuracy it can reduce forecast errors by about 40% relative to these models. Second, we undertake time-varying cross-validation tests whether the ELM model is suitable for the prediction of trend series; these tests further support the hybrid model proposed. Third, we undertake several robustness tests that suggest the main results are qualitatively very similar to changes in the length of the forecast sample. Finally, the hybrid model developed in this paper has the potential to be used in a wide range of future applications especially to series that are non-stationary and that feature complex and chaotic properties. In particular, there is scope for models featuring fuzzy entropy and extreme learning machine to be applied in many (other) areas of Management Science, Economics and Finance.

The paper has important practical and policy implications. First, the hybrid model proposed in the paper is highly advantageous in asset price prediction, which is of great significance for financial investors. The proposed model enables us to predict asset prices, as illustrated for carbon futures prices, more accurately, thus encouraging investors’ participation in asset markets and helping improve trading performance. Second, it is helpful for governments when determining the supply of carbon permits to avoid extreme levels of prices thereby facilitating the market to encourage reductions in carbon emissions. In particular the supply of carbon permits can be adjusted in response to the price levels and trend provided by the model. In addition, an interesting extension of our work would be to investigate whether forecast accuracy can be further improved by including the impact of climate change in the proposed hybrid model.

## Figures and Tables

**Fig. 14:**
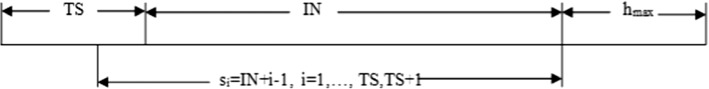
Time-varying cross-validation method

## Empirical mode decomposition (EMD)

The Empirical mode decomposition method, first proposed by Huang et al. (1998), can be used to decompose a given series into a series of intrinsic mode functions (IMFs) and a residue. The IMFs must meet the following conditions: (1) the difference between the number of extreme points and the number of zero crossings in the whole series is less than or equal to 1; (2) the mean of the upper and lower envelopes is 0 at any time. The specific decomposition steps are as follows:

*Step 1*: Find out all extreme points of time series $x(t)$, and obtain local maxima $x_{\max }$ and local minima $x_{\min }$.Then use the cubic spline interpolation method to obtain its upper and lower envelopes (i.e., $e_{\max } (t)$ and $e_{\min } (t)$). Finally, calculate the mean envelope $m(t)$ as follows: $m(t) = \frac{{e_{\min } (t) + e_{\max } (t)}}{2}$.

*Step 2*: Compute the difference between $x(t)$ and $m(t)$ to obtain $h(t)$: $h(t) = x(t) - m(t)$;

*Step 3*: Check the properties of *h(t)* whether meets the conditions of IMFs. If it meets, then *h(t)* is defined as a new IMF; otherwise, let *x(t)* = *h(t)*, return to step 1 and repeat the above steps until the conditions of IMFs are met;

*Step 4*: let the residue $r(t) = x(t) - imf(t)$;

*Step 5*: Take $r(t)$ as a new series to be decomposed, repeat the above steps until the *n-*th order IMF component $imf_{n} (t)$ or its residue $r_{n} (t)$ is either less than the default value or become a monotonic function;

Finally, *x (t)* is decomposed into the sum of all IMF components and a residue:$x(t) = \sum\limits_{i = 1}^{n} {imf_{i} (t)} + r_{n} (t)$, where *n* is the number of IMFs, and $r_{n} (t)$ is the final residue.

## ARIMA

The ARIMA model consists of auto-regressive (AR) model and moving average (MA) model. For a given time series $y_{t}$, the AR (*p*) model can be defined as:

A1 $$ y_{t} = \phi_{1} y_{t - 1} + \phi_{2} y_{t - 2} + \cdots + \phi_{p} y_{t - p} + \varepsilon_{t}$$

And the MA (*q*) model is defined as:

A2 $$ y_{t} = \varepsilon_{t} - \theta_{1} \varepsilon_{t - 1} - \theta_{2} \varepsilon_{t - 2} - \cdots - \theta_{q} \varepsilon_{t - q}$$

where the random term $\varepsilon_{t}$ is independent white noise.

So the autoregressive integrated moving average (ARIMA (*p*, *d*, *q*)) model is to combine the AR(*p*) and MA (*q*) models, where *d* is the order of the difference, which indicates that the series $y_{t}$ is a stationary series after the *d* order difference. Then the ARIMA (*p*, *d*, *q*) model can be defined as follows:

A3 $$ \phi (L)(1 - L)^{d} y_{t} = \theta (L)\varepsilon_{t}$$

where L is the lag operator, that is $L{\text{y}}_{t} = y_{t - 1}$, $\phi (L) = 1 - \phi_{1} L - \phi_{2} L^{2} - \cdots - \phi_{p} L^{p}$ and $\theta (L) = 1 - \theta_{1} L - \theta_{2} L^{2} - \cdots - \theta_{q} L^{q}$ are *p*-order, and *q*-order lag polynomials, respectively. $\phi_{i}$, $\theta_{i}$, *i* = *1, 2,…, p* are the coefficients of AR and MA models respectively, where $(1 - L)^{d}$ represents *d*-order differential operation.

## Additional evaluation criteria and loss functions

Here we present different measures to evaluate the forecasting performance of the models, including root mean square error (RMSE), mean absolute percentage error (MAPE), correlation coefficient (R) and direction statistic (DS), as the evaluation criteria. Let $y_{i}$ and $\hat{y}_{i}$ ($i=\mathrm{1,2},\dots , N$) refer to the actual and predicted values, where *N* is the sample size of the testing set. Root mean square error (RMSE) and mean absolute percentage error (MAPE) are calculated as follows:

A4 $$ RMSE = \sqrt {\frac{1}{N}\sum\limits_{i = 1}^{N} {(y_{i} - \hat{y}_{i} )^{2} } }$$

A5 $$ MAPE = \frac{100}{N}\sum\limits_{i = 1}^{N} {\left| {\frac{{y_{i} - \hat{y}_{i} }}{{y_{i} }}} \right|}$$

Correlation coefficient (R) and direction statistic (DS) are defined as follows:

A6 $$ R = \frac{{\frac{1}{N}\sum\limits_{{{\text{i}} = 1}}^{N} {(y_{i} - mean(\mathop y\limits^{\_} ))(\hat{y}_{i} - mean(\hat{y}))} }}{{\sqrt {\frac{1}{N}\sum\limits_{{{\text{i}} = 1}}^{N} {(y_{i} - mean(\mathop y\limits^{\_} ))^{2} } } *\sqrt {\frac{1}{N}\sum\limits_{i = 1}^{N} {(\hat{y}_{i} - mean(\hat{y}))^{2} } } }}$$

The correlation coefficient *R* measures the degree of linear similarity between the actual and predicted values. The larger value of R means the higher degree of collinearity between the actual and predicted values, indicating a better forecasting performance of the model. The direction statistic (DS) indicator can be used to evaluate the forecasting performance of the model from the perspective of price change directions (ups or downs). It is calculated as:

A7 $$ DS = \frac{1}{N}\sum\limits_{i = 1}^{N} {w_{i} }$$

where $w_{i}$ is as follows;

A8 $$ w_{i} = \left\{ \begin{gathered} 1,\quad if \, (y_{i + 1} - y_{i} )(\hat{y}_{i + 1} - \hat{y}_{i} ) \ge 0 \hfill \\ 0,\quad otherwise \hfill \\ \end{gathered} \right.$$

Similarly, the larger DS indicator indicates much more accurate forecasting of the direction for carbon price changes.

Moreover, when conducting the test of model confidence set (i.e., MCS) of Hansen et al. (2011) to identify the potential superior forecasting models, we use four different loss functions (i.e., *MSE, MAE, MAPE,* and *QLIKE*) as the forecasting error benchmark to compare the forecasting performance. Amon them, *RMSE* and *MAPE* are already presented as Eqs. A4 and A5, respectively; similarly, *MAE* and *QLIKE* can be expressed as follows:

A9 $$ MAE = \frac{1}{N}\sum\limits_{i = 1}^{N} {\left| {\frac{{y_{i} - \hat{y}_{i} }}{{y_{i} }}} \right|}$$

A10 $$ QLIKE = \frac{1}{N}\sum\limits_{i = 1}^{N} {(\ln \hat{y}_{i} + y_{i} /\hat{y}_{i} )}$$

where N is the number of forecasting data points.

## Time-varying cross-validation method

See Fig. ^14^.

In the traditional K-fold cross-validation method, the training sample is divided into K equal parts and then the prediction errors for all K cases are obtained. Next, the mean of K prediction errors is calculated and finally the parameter set with the smallest error is selected. However, since the traditional cross-validation method ignores the long-term memory characteristics commonly associated with carbon futures; thus this major drawback means it is not well suited to our application. Therefore, we propose a time-varying cross-validation method, and the framework of the method is shown in Fig. 14.

In Fig. 14, *TS* is the reserved sample of the model, *IN* is the first round sample used for model training, *S*_*i*_ = *IN* + *i−1* is the $i$*-*th round sample ($i=1, 2,\dots , TS$) used for model training, and *h*_*max*_ is the sample size of validation set. Therefore, an observation from the reserved sample is added to the training set in order when the cross-validation is carried out. The number of observations of training set increases in a rolling manner, and eventually the *TS* + 1 training models are obtained. In contrast to the traditional cross-validation method ignoring the serial correlation of time series, the proposed method considers the characteristics of the serial correlation instead of splitting the training sample equally. Here the sample size of the reserved sample TS and the validation set *h*_*max*_ are set to 20 and 100 (the sample size is equal to the sample size of testing set) respectively, and the range of the number of neurons in the hidden layer searched by the grid search method is set as [20, 25… 100].

## Appendix B

See Tables 13 and

**Table 14:** The identification of high/low frequency components (using fine-to-coarse reconstruction algorithms)

Contract	Stat	*IMF*_*1*_	*IMF*_*2*_	*IMF*_*3*_	*IMF*_*4*_	*IMF*_*5*_	*IMF*_6_	*IMF*_*7*_	*IMF*_*8*_	*IMF9*
Dec16	mean	0.002	0.003	0.005	−0.002	−0.009	−0.097	−0.058	−1.472	−1.832
	t	0.420	0.618	0.748	−0.200	−0.543	−4.306	−1.932	−2.927	−3.606
	P−value	0.675	0.537	0.455	0.841	0.587	**0.000**	**0.054**	**0.003**	**0.000**
Dec17	mean	0.001	0.001	0.004	−0.001	−0.001	−0.048	−0.176	0.193	0.194
	t	0.241	0.251	0.669	−0.070	−0.063	−2.785	−7.422	5.939	5.972
	P−value	0.809	0.802	0.504	0.944	0.950	**0.005**	**0.000**	**0.000**	**0.000**

Table 14 presents the identification of high−frequency and low-frequency components using the fine-to-coarse algorithm. The mean of the IMF component $Ci$ (i = 1, 2, …, 9) for the Dec16 and Dec17 is examined via t-test. As shown, starting from the C_6_ of both the Dec16 and Dec17, the mean value of the IMF series is not equal to be 0 at the significance level of 1%. Therefore, we use the fine-to-coarse algorithm to reconstruct IMF1 ~ IMF5 to the high frequency series, and reconstruct IMF6 ~ IMF9 to the low frequency series

14 and Fig. 15.

## Result of identification of ARMA model

We identify the optimal ARMA models for the high frequency series of the Dec16 and the Dec17 by the AIC criteria. The result shows that the optimal ARMA model is identified as the form of ARMA (4, 4) for the high frequency series of the Dec16. Similarly, the structure of ARMA model for the high frequency series of the Dec17 is identified as ARMA (5, 4). Further, the optimal ARMA model for the high frequency series of the training sample of the Dec16 is:

C1 $$ \begin{aligned} High_{t}  &= - 0.211High_{t - 1} + 0.782High_{t - 2} - 0.203High_{t - 3} - 0.340High_{t - 4}  \\&\quad + 0.106High_{t - 5} { + }\varepsilon_{t} + 1.243\varepsilon_{t - 2} - 0.544\varepsilon_{t - 4}  \end{aligned} $$

And the optimal ARMA model for high frequency series of the Dec17 is:

C2 $$ \begin{aligned} High_{t} & = 0.334High_{t - 1} { + }0.442High_{t - 3} - 0.659High_{t - 4} { + }0.297High_{t - 5}  \\ &\quad + \varepsilon_{t} + 0.552\varepsilon_{t - 1} + 0.545\varepsilon_{t - 2} { + }0.649\varepsilon_{t - 3} - 0.858\varepsilon_{t - 4} \hfill \\ \end{aligned}$$

Similarly, the optimal ARMA model for the low frequency series of the Dec16 is:

C3 $$ \begin{aligned} Low_{t}& = 3.123Low_{t - 1} - 4.023Low_{t - 2} { + 2}.510Low_{t - 3} { - 0}.651Low_{t - 4}  \\ &\quad + \varepsilon_{t} - 0.747\varepsilon_{t - 1} - 0.495\varepsilon_{t - 2} - 0.409\varepsilon_{t - 3} - 0.219\varepsilon_{t - 4} \\ \end{aligned} $$

And the optimal ARMA model for the low frequency series of the Dec17 is:

C4 $$ \begin{aligned} Low_{t}& = 2.323Low_{t - 1} - 1.099Low_{t - 2} - 1.606Low_{t - 3} + 2.0656Low_{t - 4}  \\ &\quad - 0.712Low_{t - 5} + \varepsilon_{t} - 2.026\varepsilon_{t - 1} - 1.854\varepsilon_{t - 2} - 1.595\varepsilon_{t - 3} - 1.306\varepsilon_{t - 4}\\&\quad - 0.726\varepsilon_{t - 5} - 0.1887\varepsilon_{t - 6}  \\ \end{aligned}$$

## Result of identification of ARMA model during the pandemic

Following the approach in Sect. 5.3, we identify the optimal ARMA models for the high and low frequency series of the Dec20 by the AIC criteria, respectively. The high and low frequency series of the training sample of the Dec20 are as follows:

C5 $$ \begin{aligned} High_{t} &= - 0.5846High_{t - 1} - 0.357High_{t - 2} + 0.329High_{t - 3} - 0.341High_{t - 4} \\&\quad - 0.131High_{t - 5} + 0.065High_{t - 6} + \varepsilon_{t} - 0.260\varepsilon_{t - 1} - 0.653\varepsilon_{t - 3} + 0.264\varepsilon_{t - 4}  \\ \end{aligned}$$

C6 $$ \begin{aligned} Low_{t} &= 4.409Low_{t - 1} - 8.604Low_{t - 2} + 9.531Low_{t - 3} - 6.343Low_{t - 4}  \\ &\quad + 2.418Low_{t - 5} - 0.417Low_{t - 6} + \varepsilon_{t} - 1.1219\varepsilon_{t - 1} - 0.253\varepsilon_{t - 2}  \\&\quad + 0.116\varepsilon_{t - 3} - 0.195\varepsilon_{t - 4}  \\ \end{aligned}$$

## Footnotes

1 Extremely low prices or excessively high prices will be problematic. Very low prices provide very little incentive for companies to reduce carbon emissions. On the other hand very high prices can place much higher costs on firms which could lead to financial distress for them.

2 Plausibly, greater predictability of carbon prices should help avoid large persistent deviations from “efficient prices” (e.g. bubbles) since market participants would notice large deviations quickly and trade accordingly to rectify this.

3 The method of empirical mode decomposition (i.e., EMD) can be found in Appendix A.1.

4 The methods of evaluation criteria of prediction performance can be found in Appendix A.3.

5 The detailed method of model confidence set (MCS) can refer to the paper of Hansen et al. (2011).

6 In the paper, we use four different loss functions (i.e., *MSE, MAE, MAPE,* and *QLIKE*) as the forecasting error benchmark. The details of loss functions can be found in Appendix A3.

7 The detailed algorithm for constructing the model confidence set (MCS) can refer to the paper of Hansen et al. (2011).

8 This approach to forecasting carbon price of futures contract is also adopted in important prior work such as Zhu and Wei (2021).

9 The lower entropy value indicates higher relevant feature with respect to the classification and contributes with more information while the higher entropy value indicates less contribution to the classification.

10 The detailed results of the identification of ARMA model can be found in Appendix C1.

11 The detailed method of time-varying cross-validation can be found in Appendix A4.

12 Our proposed hybrid method of EEMD-FuzzyEn-ARMA-ELM also enters the MCS for the Dec 20, which can be found in Table 12 in Sect. 6 of Robustness test.

13 We collect the daily prices of carbon futures contracts maturing in December 2020 (i.e., the Dec20), covering the period from January 1, 2016 to December 31, 2020. It results in a sample of total 1305 observations, which is divided into two sets, including the training set (i.e., 1044 observations) and the testing set (i.e., 261 observations), respectively.
